# sgBE: a structure-guided design of sgRNA architecture specifies base editing window and enables simultaneous conversion of cytosine and adenosine

**DOI:** 10.1186/s13059-020-02137-6

**Published:** 2020-08-28

**Authors:** Yanhong Wang, Lifang Zhou, Rui Tao, Nan Liu, Jie Long, Fengming Qin, Wenling Tang, Yang Yang, Qiang Chen, Shaohua Yao

**Affiliations:** Laboratory of Biotherapy, National Key Laboratory of Biotherapy, Cancer Center, West China Hospital, Sichuan University, Renmin Nanlu 17, Chengdu, 610041 Sichuan China

## Abstract

We present a base editing system, in which base editors are attached to different sites of sgRNA scaffold (sgBE). Each independent sgBE has its own specific editing pattern for a given target site. Among tested sgBEs, sgBE-SL4, in which deaminase is attached to the last stem-loop of sgRNA, yields the highest editing efficiency in the window several nucleotides next to the one edited by BE3. sgBE enables the simultaneous editing of adenine and cytosine. Finally, in order to facilitate in vivo base editing, we extend our sgBE system to an AAV-compatible Cas9, SaCas9 (*Staphylococcus aureus*), and observe robust base editing.

## Introduction

The clustered regularly interspaced short palindromic repeats (CRISPR)-based genome editing tools have shown significant successes in basic biomedical research and also provided great promise in clinical translation [1–3]. Recently, the development of base editing technique has enabled the conversion of one target DNA base pairs to another (C/G➔T/A or A/T➔G/C) in an efficient and irreversible way without causing robust double-strand breaks (DSBs), which significantly reduced off-target effects [4, 5] and made it practicable to correct genetic lesions of point mutation in inherited diseases [3].

Basically, base editors consist of a catalytically impaired Cas9 protein tethered with cytidine or adenosine deaminases that are active on ssDNA substrates. Cas9 binds a genomic locus of interest through the guidance of single sgRNA to form a protein-RNA-DNA ternary “R-loop” complex, in which the nontarget strand of sgRNA (NTS) is partially detached from the complex [6]. The exposed NTS provided a feasible substrate for deaminases to action. The most frequent way to tether deaminases is fusing them, through various linkers, to the N-terminus of Cas9 protein [4, 7]. Base editors (BEs) with such architecture, including BE3, BE4, and ABE7.10, usually catalyzes the conversion of bases in a special region within NTS, called editing window, which is ~ 5 nt in width and ~ 15 nt upstream the protospacer adjacent motif (PAM) [2, 4, 5].

Although base editing technology has achieved significant success in a variety of basic biomedical researches [8–11], there are several limitations of current base editors standing in the way to clinical translation. In particular, the fixed editing windows of current base editors and definite PAMs of Cas9s employed in these editors significantly limited their targetable sites [12]. Besides, current base editors are too large to be in vivo delivered by adeno-associated virus (AAV), one of the most efficient gene therapy vectors [13, 14]. Therefore, there is a growing need for diversifying base editing tools capable of making designed changes in any given position and compatible with AAV delivery system.

Here, we sought to construct a base editing system in which deaminases were attached to the Cas9 complex through the interaction of MS2 RNA and its binding protein, MS2 coating protein (MCP) [15–17]. Previous studies had revealed that sgRNAs tagged with MS2 RNA were able to recruit various modulators to the Cas9 complex, such as transcriptional regulators, epigenetic modifiers, and cytosine deaminases [18–20]. In this platform, MS2 RNA tags are fused to sgRNA on its 1st and 3rd stem-loops, whereby they recruit these modulators to the Cas9 complex through fusing them to MCP. While this platform is efficient in a wide range of actions, including transcriptional regulation and visualization of local chromatin, it was less efficient in base editing [19]. This is possibly due to the fact that unlike other systems, the base editing system requires direct interaction between deaminases and their substrate, the NTS. Therefore, the relative location of deaminases to NTS is a key factor determining the scope and efficiency of base editing. According to a recently published Cryo-EM structure of the Cas9/DNA/sgRNA complex (PDB 5y36) [21], which for the first time presented the scenario with detached NTS included, each stem-loop of sgRNA scaffold had distinct positions relative to NTS. We designed a series of base editors in which deaminases were tethered to different sites of sgRNA scaffold and thereby were placed to different locations relative to NTS. In a set of target sites, these base editors generated distinct editing patterns. Importantly, one of these editors, sgCBE-SL4, in which deaminase was attached to the last stem-loop of sgRNA, yielded comparable editing efficiency to BE3, the most popular base editor, with an editing window several nucleotides next to the one edited by BE3s. By extending the sgBE with other Cas9 protein and cytosine deaminases, we further expanded the sgBE toolbox with AAV compatibility and diversified editing window.

## Results

### Design and characterization of cytosine base editor with MS2-MCP-tethered APOBEC1 cytosine deaminase (sgCBE)

**Fig. 1:**
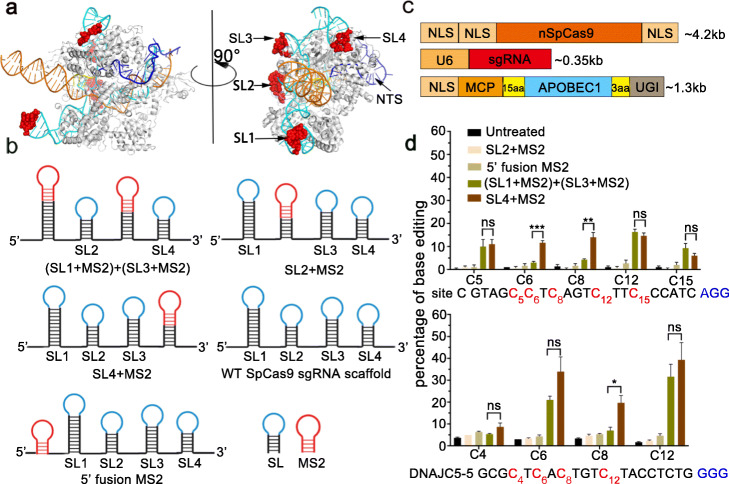
Base editing of genomic DNA in human cells by sgCBE. **a** Cartoon representation of the Cryo-EM structure of SpyCas9 in complex with DNA and sgRNA. Loops of SpyCas9 sgRNA for inserting MS2 tag were shown in red spheres. **b** Sequence of SpCas9 and schematic diagram of MS2-modified sgRNA scaffold. **c** Organization of sgRNA-derived cytosine base editor (sgCBE). SpyCas9 D10A nickase, APOBEC1, and MS2-sgRNA were expressed separately. The cytosine deaminase, APOBEC1, was fused with MCP and UGI in its N-terminus and C-terminus, respectively, to form MCP-APOBEC1. **d** Efficiency of cytosine editing with various MS2-sgRNA-derived base editors. HEK293T cells were transfected with plasmids harboring SpCas9 nickase, MCP-APOBEC1, and different MS2-modified sgRNA to form sgCBE. Target Cs were shown in red, with a subscripted number denoting their relative position to PAM (counting NGG PAM as + 21 to + 23), and the PAM sequence was shown in blue. C-to-T editing efficiencies were analyzed by Sanger sequencing and EditR calculating. Each experiment was repeated at least three times. Data are represented as mean ± SEM; ^ns^*p* > 0.05, *p < 0.05, **p < 0.01, ***p < 0.001

To test our hypothesis that tethering of deaminase onto sites close to the NTS would increase the editing efficiency and expand the window, we searched for candidate tethering sites by analyzing the 3D structure of the Cryo-EM structure of the Cas9/DNA/sgRNA complex [21]. We found that the 5′ spacer region and the 4th stem-loop were much closer to the NTS than the 1st or the 3rd stem-loop. The 2nd stem-loop located on the other side opposite to NTS was even more distant to the NTS than the 1st or the 3rd stem-loop (Fig. 1a).

We fused MS2 RNA tag to these sites respectively to generate a series of MS2 tagged sgRNAs, including SL2+MS2, SL4+MS2, 5′ fusion MS2, and (SL1+MS2)+(SL3+MS2) sgRNAs (Fig. 1b). Then, we fused cytosine deaminase, APOBEC1, and uracil-DNA glycosidase inhibitor (UGI) with MS2 RNA-interacting protein (MCP) (Fig. 1c). A previously developed base editing tool, CRISPR-X [19], used (SL1+MS2)+(SL3+MS2) sgRNA recruit AID to dead SpCas9 (dSpCas9) but showed very limited activity, which was possibly due to dSpCas9 lacking the activity to nick the target DNA strand. We compared the activities of dCas9 and nickase SpCas9 (nSpCas9) in the case of (SL1+MS2)+(SL3+MS2) sgRNA. As expected, nSpCas9 performed much better than dSpCas9, as detected by Sanger sequencing and subsequent EditR analysis (Additional file 1: Fig. S1) [22]. EditR is a calculating program developed to identify and quantify base editing from fluorescent Sanger sequencing paragraph. Due to the presence of baseline noise in Sanger sequencing results, the detection limit for EditR program is usually above 5%. Therefore, only efficiencies above 5% were considered to have been effectively edited.

Next, we tested the performance of those newly designed sgRNAs. Co-transfection of these MS2-sgRNA, MCP-APOBEC1, and nickase SpCas9 constructs revealed that only SL4+MS2 and (SL1+MS2)+(SL3+MS2) sgRNAs produced obvious base editing. As shown in Fig. 1d, neither 5′ fusion MS2 nor SL2+MS2 sgRNA produced detectable editing. Compared to the most popular MS2-sgRNA, (SL1+MS2)+(SL3+MS2) sgRNA and SL4+MS2 sgRNA produced much more robust editing in both sites tested. On average, editing efficiency in each targetable cytosines by SL4+MS2 sgRNA was ~ 40% higher than that by (SL1+MS2)+(SL3+MS2) sgRNA (16.14% vs 11.55%) (Fig. 1d and Additional file 1: Fig. S2), suggesting that reducing the distance between deaminase and the NTS would increase the editing efficiency.

In Fig. 1d, we also noticed that for (SL1+MS2)+(SL3+MS2) sgRNA, the editing efficiency in each different cytosines significantly varied. It seemed that (SL1+MS2)+(SL3+MS2) sgRNA had two editing peaks, one around position 5 and the other around position 12 (counting NGG PAM as 21–23), and cytosines in-between these two peaks were less efficiently edited. The variation in editing efficiency of each position was unlikely due to motif preference of the APOBEC1, because SL4sgRNA produced extensive editing in those positions that were poorly edited by (SL1+MS2)+(SL3+MS2) sgRNA.

### A systematic examination of the effects of tethering sites on base editing behaviors

**Fig. 2:**
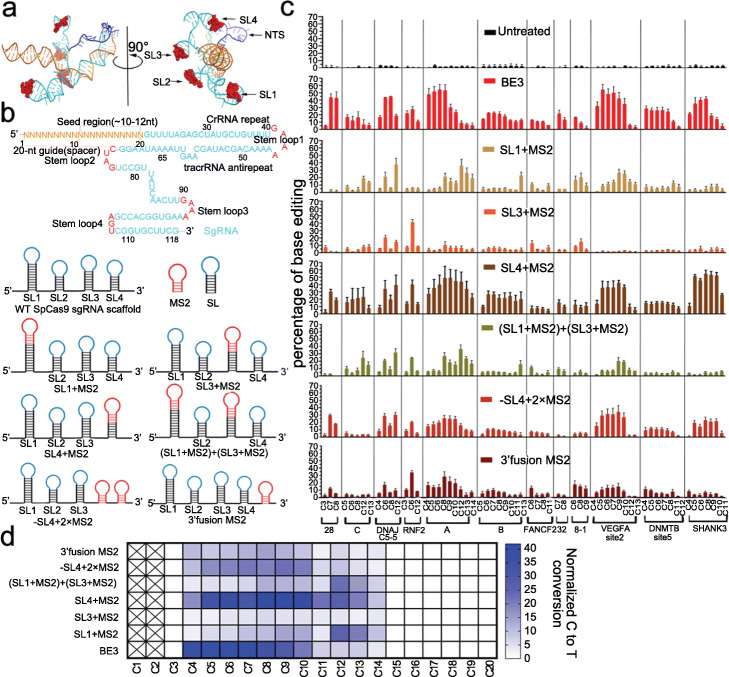
Characterization of sgCBEs derived from various MS2 sgRNAs. **a** Cartoon representation of the Cryo-EM structure of SpCas9 sgRNA and NTS. The location of each loop of sgRNA was shown in red. **b** SpCas9 sgRNA sequence and schematic diagram of MS2-modified sgRNA scaffold. **c** Efficiency of cytosine editing with various MS2-sgRNA derived base editors. HEK293T cells were transfected with plasmids expressing BE3 or sgCBE that targeted a set of 12 different sites. C-to-T editing efficiencies were analyzed by Sanger sequencing and EditR calculating. Each experiment was repeated at least three times. Data are represented as mean ± SEM. **d** Heat map showing the base editing window of various base editors. Four sites that harbored multiple Cs, including site A, site B, VEGFA site2, and SHANK3, were used to summarize the editing window, and the average C-to-T conversion rate of the indicated potion was calculated. Each experiment was repeated at least three times. Data are represented as mean ± SEM

Inspired by the observation that tethering deaminase to different stem-loops of SpCas9 sgRNA endowed base editors with different editing patterns (editing efficiency and window), we systematically designed a series of MS2-sgRNAs, in which MS2 was fused to different stem-loops to form additional MS2-sgRNAs, including SL1+MS2, SL3+MS2, -SL4+2×MS2, and 3′ fusion MS2 (Fig. 2a, b). We then co-transfected those MS2-sgRNAs targeting a set of 12 sites together with SpCas9 nickase and MCP-APOBEC1 constructs into HEK293T cells and examined the editing patterns of each MS2-sgRNA by Sanger sequencing and EditR analysis. In consistence with previous observations, MS2 tagged to SL4 or SL4+MS2 sgRNA had the most efficient base editing that was close to the level of BE3.

Besides overall editing activity, we noticed that for a given target, those sgCBEs generated different editing patterns (editing window and editing efficiency in each cytosine), all of which are quite different from the ones generated by BE3, the most popular base editing tool (Fig. 2c and Additional file 1: Fig. S3). Generally, BE3 showed an editing window ranging from C3 to C8, with peak positions at C5 to C7 [4]. Compared to BE3, SL4+MS2 sgRNA produced a slightly wider editing window, with peak positions at C5 to C10. The editing windows of -SL4+2×MS2 and 3′ fusion MS2 were similar to that of SL4+MS2 sgRNA, yet with lower efficiencies (Fig. 2c, d). SL1+MS2 and (SL1+MS2)+(SL3+MS2) showed irregular editing windows that were narrower than those of BE3 or SL4+MS2. The editing patterns of SL1+MS2 and (SL1+MS2)+(SL3+MS2) were similar on most target sites except for site 28. On this site, SL1+MS2 had very limited activity. Interestingly, on the same site, SL3+MS2 had similar editing activity and pattern to (SL1+MS2)+(SL3+MS2), suggesting that both SL1 and SL3 contributed to the action of (SL1+MS2)+(SL3+MS2). Together with the observations in Fig. 1d, these data confirmed the notion that different tethering sites of deaminase produced different editing patterns, which was particularly meaningful in situations where the target cytosines were located in complicated circumstances.

To confirm the accuracy of the above data determined by EditR, we conducted a high-throughput sequencing analysis on a set of 5 sites, including sites 28, 30, C, DNAJC5-5, and RNF2. In agreement with a previous report [22], the results of HTS were consistent with those obtained from EditR analysis (Additional file 1: Fig. S4). Next, we sought to replace MS2-MCP with another RNA-protein interaction system, BoxB-λN22 [23]. We found that sgCBEs modified with BoxB had a similar editing pattern and activity to the ones modified with MS2 (Additional file 1: Fig. S5).

Base editing involves C to U conversion in the NTS and ssDNA strand break in the target strand (TS), and those genomic lesions are fixed by the DNA repair system, occasionally resulting in indel formation and base conversions besides C to T (C to R, R=A or G). Next, we sought to study the purity of C to T conversions in the editing products of the above 5 sites that had been examined by HTS. The HTS analysis revealed that all base editors induced indel formations across 5 sites, and sgCBEs induced less indels than BE3 did in 4 out of 5 sites (Additional file 1: Fig. S6). The sgCBEs also induced less C to R conversions than BE3 (Additional file 1: Fig. S6). These results suggested that sgCBEs performed better than BE3 in terms of product purity.

Previous studies have found that CBEs have off-target DNA editing in both sequence-dependent and sequence-independent manners. To define the specificity of sgCBEs, 3 on-target sites were selected for sequence-dependent off-target analysis. A total of 11 off-target sites (OTSs) were predicted with an online program (Table S5), all of which contained Cs within the editing window of BE3 or sgCBEs. HTS results showed that in the majority of OTSs, the mutation rates of OTS induced by sgCBEs were comparable to those induced by BE3(Additional file 1: Fig. S7).

### Diversify the sgCBE editing tools with additional cytosine deaminases

Several eukaryote-derived cytosine deaminases had been demonstrated efficient in base editing, including CDA, AID, Target-AID, A3A, and APOBEC1 [4, 7, 24–26]. Among them, APOBEC1 from rat was most frequently used. Although there were very limited data of comparing the editing efficiency and scope between APOBEC1 and the rest deaminases, two recent reports suggested that at least A3A had different editing patterns to APOBEC1 [25, 27], even under the same settings, such as BE3 architecture which included a 16aa XTEN linker between deaminase and Cas9 protein plus one copy of UGI fused to the C-terminus of Cas9.

**Fig. 3:**
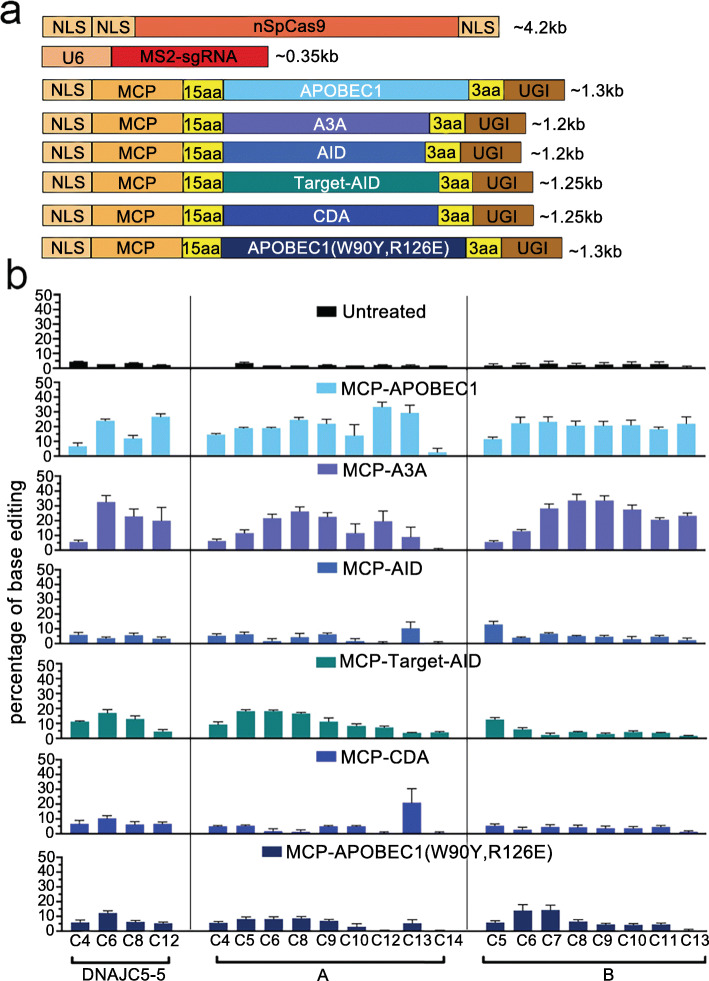
Comparison between base editing of APOBEC1-derived sgCBE and other deaminase-derived sgCBEs. **a** Architectures of sgCBEs. **b** Efficiency of cytosine editing with sgCBE derived from various cytosine deaminases. HEK293T cells were transfected with various sgCBEs targeting a set of three different sites. C-to-T editing efficiencies were analyzed by Sanger sequencing and EditR calculating. Each experiment was repeated at least three times. Data are represented as mean ± SEM

We investigated if we could further obtain additional pattern-specified editing tools, simply by changing the cytosine deaminases in our system, including A3A, AID, CDA, and Target-AID. When coupled with these deaminases to sgRNAs, through the interaction between SL4+MS2 and MCP, only A3A displayed comparable editing activity to APOBEC1. AID, Target-AID, and CDA did not generate robust base editing. Interestingly, in all the 3 target sites tested, A3A produced distinct editing patterns from APOBEC1 (Fig. 3b and Additional file 1: Fig. S8). Although both of them displayed a wide editing window, from C4 to C13, A3A seemed to prefer the cytosines located in the 5′ portions of this window, as compared to APOBEC1. Then, we compared the editing patterns between APOBEC1 and A3A in other MS2-sgRNA settings. In consistence with the observations in SL4+MS2, A3A displayed different editing patterns from APOBEC1 when they were coupled to those MS2-sgRNAs (Additional file 1: Fig. S9). Together, these results suggested that the substitution of APOBEC1 with A3A further diversified the editing pattern of sgCBE.

We also tested if we could narrow the editing window of each MS2-sgRNA by impairing the cytosine activity. We introduced 2 point mutations, W90Y and R126E, which had been previously demonstrated to be able to narrow the editing window of BE3-APOBEC1 [28, 29]. As shown in Fig. 3b, mutant APOBEC1 did mildly narrow the editing window in 2 out of 3 sites (site DNAJC5-5 and site B), albeit at the expense of lowering editing efficiency.

### Design and characterization of adenosine base editor based on sgRNA-MS2/MCP system (sgABE)

**Fig. 4:**
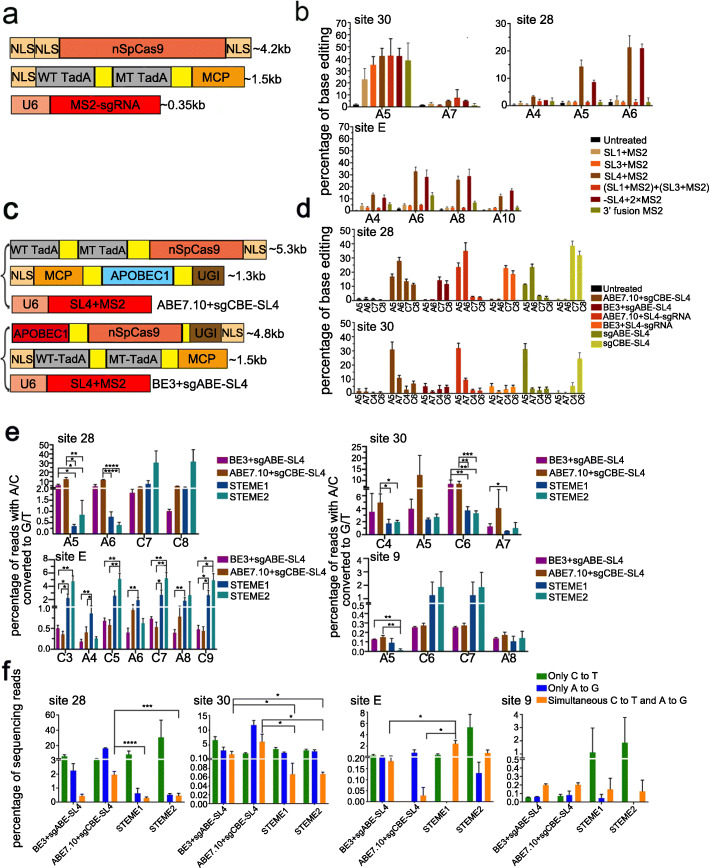
Simultaneous editing of adenine and cytosine by sgBE. **a** Architecture of sgABE. **b** Efficiency of adenine editing with sgABEs. HEK293T cells were transfected with plasmids expressing sgABEs targeting a set of three different sites. A-to-G editing efficiencies were analyzed by Sanger sequencing and EditR calculating. Each experiment was repeated at least three times. Data are represented as mean ± SEM. **c** Architectures showing different combinations of ABE+CBE. **d** Efficiency of cytosine or/and adenosine editing with different ABE+CBE combinations or single ABE/BEs. HEK293T cells were transfected with plasmids expressed indicated base editors or their combinations. C-to-T and A-to-G editing efficiencies were analyzed by Sanger sequencing and EditR calculating. Each experiment was repeated at least three times. Data are represented as mean ± SEM. **e** Efficiency of cytosine or/and adenosine editing with different ABE+CBE combinations and STEME systems. C-to-T and A-to-G editing efficiencies were analyzed by deep sequencing. Each experiment was repeated at least three times. Data are represented mean ± SEM; ^ns^*p* > 0.05, *p < 0.05, **p < 0.01, ***p < 0.001. **f** The ratio of single and simultaneous editing products generated by different ABE+CBE combinations and STEME systems at 4 target sites. Each experiment was repeated at least three times. Data were represented as mean ± SEM; ^ns^*p* > 0.05, *p < 0.05, **p < 0.01, ***p < 0.001

After establishing the sgCBE system, we investigated if we could apply our MS2-sgRNA system to adenosine base editing (sgABE). We fused the ABE editor, the hetero-dimer of a wild-type TadA (WT TadA) and an evolved mutant TadA (MT TadA), to the N-terminus of MCP with a 32-aa linker to form TadA-MCP. Then, we co-transfected this construct together with a set of MS2-sgRNAs harboring varied MS2 fusion and different spacers. In all three targets tested, SL4-derived sgABEs, including SL4+MS2 and -SL4+2×MS2, produced robust adenosine conversion (Fig. 4b and Additional file 1: Fig. S10). The rest of the sgABEs only produced robust adenosine editing in one target. These data were consistent with the observations in sgCBE, in which SL4-derived editors were more efficient than that derived from other SLs.

After establishing both sgCBE and sgABE systems, we sought to combine ABE7.10 or BE3 with our sgBEs to form dual base editors capable of simultaneously converting both cytosines and adenosines in one target site (Fig. 4c). Co-transfection of ABE7.10 with SL4+MS2-derived sgCBE (sgCBE-SL4) generated detectable conversion of both bases in both target sites as determined by Sanger sequencing and EditR analysis, while the combination of BE3 and sgABE-SL4 did not (Fig. 4d and Additional file 1: Fig. S11). In comparison between the combinations (ABE7.10+sgCBE-SL4 or BE3+sgABE-SL4) and single-base editors (ABE7.10-SL4, BE3-SL4, sgABE-SL4, or sgCBE-SL4), we noticed that the combination reduced the editing efficiency of both types of editors. For example, in site 28, the combination of ABE7.10 and sgCBE-SL4 resulted in a 23.3% loss of adenosine editing compared with ABE7.10+SL4-sgRNA and 64.62% loss of cytosine editing compared with sgCBE-SL4. In the same site, the combination of BE3 and sgABE-SL4 also obviously reduced both cytosine and adenosine editing (37% loss for cytosine and nearly 100% loss for adenosine, as compared to BE3 and sgABE-SL4, respectively). These results suggested when targeting the same site, ABE and CBE might interrupt each other, which was possibly due to the fact that both editors could bind to ssDNA and thus competed with each other when they were put in the same target site. In consistence with our observations, a recent report using TadA and A3A fusion editors also observed dramatic inhibition of TadA activity [30].

In addition, we designed dual tag sgBEs using MS2 and BoxB RNA scaffolds to recruit cytosine and adenine deaminases (Additional file 1: Fig. S12). However, compared to the original strategy, dual tag sgBEs produced much lower editing (Fig. S12). Then, we focused on the original dual base editors for further characterization. To analyze the efficiency of simultaneous conversion, HTS was performed and additional targets were examined. The results showed that our dual base editors produced simultaneous conversion of A and C across all 4 targets, with an efficiency ranging from 0.2 to 8% (Fig. 4e, f). We also compared our dual base editors with previously published STEME in plants. In comparison, we found that all those dual base editors produced simultaneous A and C editing, yet the efficiency of which varied greatly dependent on target sites tested and the editors used (Fig 4e, f). Overall, our dual base editors were less efficient than STEME in terms of C-to-T editing, but their activity of A-to-G and simultaneous editing was similar to or higher than STEME (Fig. 4e, f).

### Expanding sgBE to SaCas9, an AAV-compatible CRISPR system

A critical obstacle towards the way of base editing to the therapeutic application is the large size of current base editors [14], especially SpCas9-derived base editors. Compared to SpCas9, SaCas9 has a much smaller size, ~ 3.2 kb, which makes it more commonly used than SpCas9 in in vivo delivery for therapeutic purposes, especially through the AAV delivery system [31, 32]. Therefore, developing a SaCas9-based sgBE system (Sa-sgCBE) is meaningful for translational research of base editing technology.

**Fig. 5:**
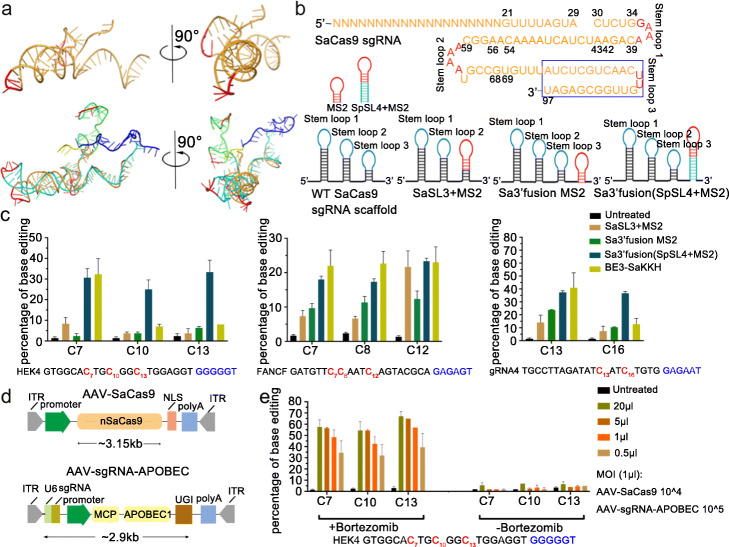
Base editing of genomic DNA in human cells by Sa-sgCBE. **a** Cartoon representation of the comparison between SpCas9 sgRNA and SaCas9 sgRNAs. The upper cartoons were the crystal structure of the SaCas9 sgRNA scaffold. Below cartoons represented the comparison between the SaCas9 sgRNA scaffold and the SpCas9 sgRNA scaffold. Loops of each sgRNAs were shown in red. **b** SaCas9 sgRNA sequence and schematic diagram of MS2-modified sgRNA scaffold. (Blue box enclosed the 3rd stem-loop that was deleted when resolving the crystal structure). **c** Efficiency of cytosine editing with Sa-sgCBE. HEK293T cells were transfected with plasmids expressing BE3-SaKKH or Sa-sgCBEs targeting a set of three different sites. C-to-T editing efficiencies were analyzed by Sanger sequencing and EditR calculating. Each experiment was repeated at least three times. **d** Schematic diagram showing AAV vectors encoding Sa-sgCBE, one expressing SaCas9 nickase and the other one expressing U6-sgRNA and MCP-APOBEC1-UGI. **e** Efficiency of cytosine editing with AAV-encoded Sa-sgCBE. HEK293T cells were transduced with Sa-sgCBE AAVs targeting HEK4 site at the indicated multiplicity of infection (MOI), with or without bortezomib treatment. Four days later, cells were harvested and subjected to DNA analysis

Because there is no available SaCas9 structure that included the NTS, we sought to design Sa-sgCBE following the parameters obtained from SpCas9. Firstly, we compared the structure of the SaCas9 complex (PDB:5axw) [33] with the one of SpCas9 with NTS included (PDB:5y36) [21]. In comparison, we found that although these Cas9s shared only 17% identical amino acids, their general structures were largely alike. The positions of sgRNA, the PAM region of the target DNA, the sgRNA/TS heteroduplex, and various Cas9 domains in the SaCas9 complex were almost the same as those in SpCas9 (Additional file 1: Fig. S13). Importantly, the shape of SaCas9 sgRNA, especially the direction of its stem-loops, was also similar to that of SpCas9 sgRNA. However, the SaCas9 complex used to resolve the structure only included an sgRNA with 2 stem-loops, which lacked the 3rd stem-loop as compared to the ones that were frequently used in SaCas9-mediated gene editing experiments (Fig. 5a). A previous study in SpCas9 had revealed that sgRNAs with 3′ stem-loop deleted would decrease gene editing activity [34]; therefore, we chose the 3-stem/loop SaCas9 sgRNA for subsequent designs.

Following the guidance of the architecture of SpCas9 SL4+MS2 sgRNA, which is the most efficient one, we designed a series of MS2-Sa-sgRNAs, including SaSL3+MS2, Sa 3’fusion MS2, and Sa 3’fusion (SpSL4+MS2) (Fig. 5b). Transfection of these MS2-Sa-sgRNAs that harbored various spacers revealed that all these sgRNAs produced detectable base editing, with an activity order: Sa3’fusion (SpSL4+MS2) >Sa3’fusion MS2>SaSL3+MS2 (Fig. 5c and Additional file 1: Fig. S14). Notably, Sa3’fusion (SpSL4+MS2) sgRNA-derived cytosine editor (Sa-sgBE-SpSL4) had a similar editing activity to BE3-SaCas9, with a 3′ expanded editing window, which was in consistence with the observations in SpCas9-derived sgCBE-SL4. Next, in order to prove the notion that Sa-sgBE was compatible for AAV delivery, we constructed its elements into two AAV vectors (Fig. 5d), one expressing SaCas9 nickase and the other expressing MCP-APOBEC1-UGI and sgRNA. The resulting AAVs were used to transduce 293T cells. To improve the transduction, cells were treated with bortezomib, a commonly utilized proteasome inhibitor that was known to improve AAV transduction in multiple cell types [35]. As shown in Fig. 5e, our Sa-sgBE could be efficiently expressed by AAVs and produced robust base editing in 293 cells in the presence of bortezomib. In the absence of bortezomib, editing activity was significantly reduced, suggesting the improvement of AAV transduction by proteasome inhibition.

## Discussion

Current base editors, in which deaminases were fused to the N-terminus of Cas9, such as BE3 and ABE7.10 [4, 5], catalyze the conversion of bases in an ~ 5-nt window that is ~ 15 nt upstream the PAM. This editing window is a double-edged sword. On one hand, widening the editing window will increase the targetable range. However, the expansion of the editing window will also increase the probability to introduce unwanted editing, which sometimes discounted or even disabled the aimed editing. On the other hand, narrowing the window will increase the editing precision while also lead to the inability to the target sites without a proper neighboring PAM. An ideal way to resolve this obstacle is to expand base editing toolbox with editors possessing diversified editing windows, especially considering those targets that were located in a complicated circumstance. These diversified editing tools would make it possible to optimize the editing strategy for the complicated targets.

Previous studies had demonstrated that translocation of deaminases by directly inlaying them into various internal sites of Cas9 protein lead to varied editing window [36, 37]. Although these strategies more or less impaired the editing activity, possibly due to the interruption of Cas9 integrity, they proved the concept that positioning deaminases to different regions of the RNP complex would affect the editing window. As mentioned above, according to the Cryo-EM structure of the SpCas9 RNP complex, each stem-loop of sgRNA had different localizations relative to NTS, which provided a possibility to design editing tools possessing varied editing window while keeping Cas9 intact. A systematic attachment of cytosine deaminase to all stem-loops revealed that SL1, SL3, and SL4 generated robust base editing. Importantly, these CBEs also had different editing windows. Therefore, these editors provided a possibility for optimizing editing strategy for some complicated targets. When wide editing range is required, such as mutating gene enhancers, SL4 may be a good choice, since it has a wider editing window. And when a single base is aimed and this base is surrounded by other editable bases, these editors could be screened for improving the relative editing of target base to unwanted bases. Taken site B locating at *DNMT3B* locus as an example (Additional file 1: Fig. S15), if the 438th amino acid is required to be converted from glutamic acid to lysine while keeping other potions unchanged, SL1 and SL1+SL3 would be the best choice. These data also proved a notion that reengineering the appending architecture of sgRNA scaffold could generate base editors with distinct editing features. Considering that the scaffold modifications described in this study are only simply grafting MS2 to different stem-loops of sgRNAs, there is still room for designing more complicated and precise architectures to improve this toolbox. For example, the appending architectures can be designed to place MS2 or other tags to the site close to a specific NTS fragment, further improving the precision of the editing window. In addition, sgRNA architectures can also be designed to carry multiple RNA tags, such as PP7 and S1 [38, 39], which in turn recruit multiple players to perform complicated reactions, such as modulating DNA repair programs following base deamination. It should be noted that off-target editing of base editors has been observed in both RNA and DNA [40, 41]. Adenosine base editors induced mainly RNA off-target mutations, and cytosine base editors induced both RNA and DNA off-target mutations. Although we did not examine RNA and sequence-independent DNA off-target editing, considering that deaminases were being separately expressed and would be leaked from the binding of the Cas9 complex, sgBEs might have higher off-target editing of both types than BE3. Recently, novel versions of cytosine and adenosine deaminases with lower off-target editing were developed [42, 43], which provide a possible solution for reducing sgBEs’ off-target editing.

In addition, sgBE system provided a strategy to simultaneously convert both adenosines and cytosines in a given window of the genome, which is particularly useful in conditions where a specific mosaic mutant pattern was required, especially those aimed to change bases within a small window, say within a 3–5-bp window. Besides, simultaneous conversion of adenosines and cytosines also makes base editing more powerful for the unbiased screening that targets a relatively limited DNA fragment, for example, the evolution of the antibody or fluorescence protein.

Finally, the sgBE system also provided a solution for in vivo delivery of base editors by AAV vectors, the currently most efficient in vivo delivery tool. The major obstacle for the in vivo applications of current base editors is its big size (~ 5.3 kb for SpCas9 and ~ 4.2 kb for SaCas9-derived editors), which is larger than or very close to the package limitation of AAV. One possible way to deliver such base editors with AAV is to split the base editors into two parts. This strategy often involves the split of Cas9 proteins into two expression cassettes and reconstituted by intein-mediated protein splicing [44–48], which potentially attenuated the editing efficiency. Our sgBE system does not require the split of Cas9 proteins and thus generates a similar editing efficiency as that of corresponding full-length base editors. The sgBE system can be in vivo delivered by two AAV vectors, one harboring Cas9 nickase cassette and the other harboring MCP-deaminase plus sgRNA cassettes.

## Conclusion

We engineered the sgRNA scaffold of SpCas9 and used RNA/RNP interaction system to recruit cytosine or adenosine deaminases. The resulting sgBEs had diversified editing patterns, all of which were distinct to that of BE3. When coupled with traditional CBE or ABE, sgBEs enabled simultaneously conversion of both cytosine and adenosine in a single target. In addition, we also expanded these findings to SaCas9 to generate an AAV-compatible sgBE. In summary, this study developed a series of base editors with diverse editing features, which offer an opportunity for the optimization of editing tools when the target bases were located in complicated circumstances, and also provided a base editing tool for in vivo gene corrections.

## Materials and methods

### Plasmid construction

Plasmids encoding BE3, BE3-SaKKH (SaCas9 KKH variant), and ABE7.10 editors were obtained from addgene (#73021, #85170, #102919). The scaffold of the sgRNA tagged with MS2 or BoxB was synthesized by General Biosystems (Anhui) Co. Ltd. Sequences for each MS2-sgRNAs or BoxB-sgRNAs were listed in the Note S1. The corresponding spacers were inserted into each sgRNAs digested with Bbs1. Oligos used to generate sgRNA plasmids were listed in Additional file 1: Table S1. The rest of the plasmids, including MCP-deaminases, λN22-deaminases,, pAAV-CMV-saKKH (D10A), and pAAV-U6sgRNA-CMV-MCP-APOBEC1-UGI were constructed through the seamless cloning method (ClonExpress II One Step Cloning Kit. Vazyme Biotech Co. Ltd). All plasmids were verified by Sanger sequencing.

### Cell culture

HEK293T were cultured in Dulbecco’s modified Eagle’s medium (Thermo Fisher Scientific), supplemented with 10% (v/v) fetal bovine serum (Life Technologies) and 1% penicillin/streptomycin (Boster Biological Technology Co. Ltd.), and maintained at 37 °C with 5% CO_2_.

### Plasmid transfection and AAV transduction

HEK293T cells were seeded on 96-well plates (BIOFIL). Cells at a confluence of ~ 70–80% were transfected with Transeasy™ (Forgene) according to the manufacturer’s instruction. Seventy-two hours post-transfection, genomic DNA was extracted by the addition of 30 μl freshly prepared lysis buffer. The mixture was incubated at 55 °C for 10 min and then was heat-inactivated at 95 °C for another 10 min. The resulting DNA lysate was subjected to PCR amplification and subsequent analysis.

For packaging serotype 2 AAVs, pAAV-CMV-SaKKH (D10A) or pAAV-CMV-MCP-APOBEC1-U6sgRNA were co-transfected with pHelper and AAV2 Rep-Cap plasmids into HEK293 cells. The resulting AAVs were extracted from host cells by three cycles of freeze-thawing, then were purified by ultra-centrifuge. AAV titers were determined by qPCR with a pair of primers targeting CMV promoter, CMV forward: 5′CTGACCGCCCAACGACCC, CMV reverse: 5′CTGACCGCCCAACGACCC. For AAV transduction assay, cells at a confluence of ~ 70–80% were transduced with AAV2-CMV-SaKKH(D10A) and pAAV2-CMV-MCP-APOBEC1-U6sgRNA. Sixty-five nanomole bortezomib was added to the culture medium to increase AAV transduction as previously described [35]. Four days later, AAV-transduced cells were harvested for DNA analysis.

### Base editing analysis with Sanger sequencing and EditR software

On-target genomic regions of interest were amplified by PCR and then were analyzed with Sanger sequencing. Then, the sequencing graphs were further quantified by EditR software (baseditr.com), according to the author’s description. Primers used for amplifying each target loci are listed in Table S2.

### High-throughput sequencing of genomic DNA samples

Cells were harvested at 72 h post-transfection, and the genomic DNA was extracted with freshly prepared lysis buffer. Genomic regions of interest were amplified by PCR with primers flanked with different barcodes (Table S3 and S4). The products were purified with DNA gel-extraction kit and quantified with NanoDrop (Thermo Fisher). Samples were sequenced commercially using the Illumina Novaseq6000 platform (Tsingke, Beijing and Personal Biotechnology, Shanghai, China).

## Supplementary information

**Additional file 1.** Integrated supplementary Figures, Tables and Note. Contains figures from S1 to S15, tables from S1 to S5 and Note S1.

**Additional file 2.** Review history.

## Data Availability

Deep-sequencing data are available under BioProject ID PRJNA655177 (https://www.ncbi.nlm.nih.gov/bioproject/PRJNA655177) [49].
