# Supplementary material for: sgBE: a structure-guided design of sgRNA architecture specifies base editing window and enables simultaneous conversion of cytosine and adenosine
Source: Genome Biol. 2020 Aug 28;21:222. doi: 10.1186/s13059-020-02137-6 (PMC7453718; doi:10.1186/s13059-020-02137-6)
Supplement: Supplementary file 1 — Additional file 1. Integrated supplementary Figures, Tables and Note. Contains figures from S1 to S15, tables from S1 to S5 and Note S1. [file 13059_2020_2137_MOESM1_ESM.docx]

**Supplementary information**

**sgBE: a structure-guided design of sgRNA architecture specifies base editing window and enables simultaneous conversion of cytosine and adenosine**


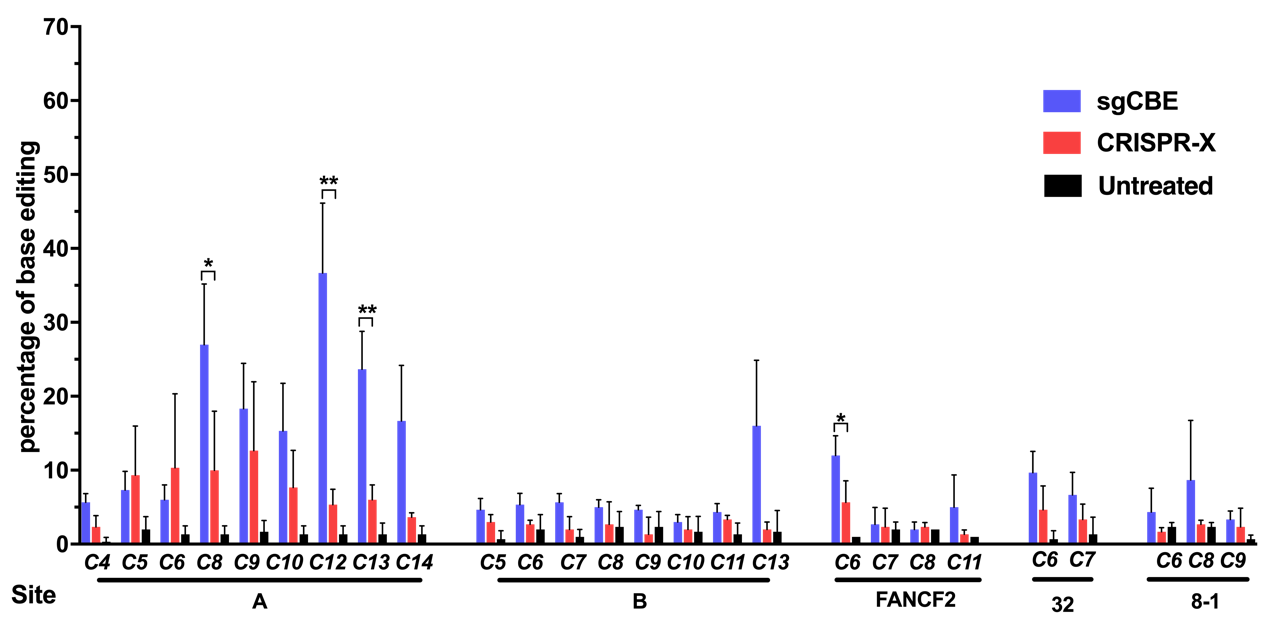


**Figure S1. Base editing of sgCBE and CRISPR-X**. Five target sites were tested to compare (SL1+MS2)+(SL3+MS2) sgCBE with CRISPR-X. HEK293T cells were transfected with (SL1+MS2)+(SL3+MS2) sgCBE or CRISPR-X, targeting siteA, siteB, FANCF2, site32 and site8-1. All the sites were amplified by PCR and the resulting products were subjected to Sanger sequencing and EditR analysis. Each experiment was repeated at least three times, data are represented mean ± SEM; *p<0.05, **p<0.01.


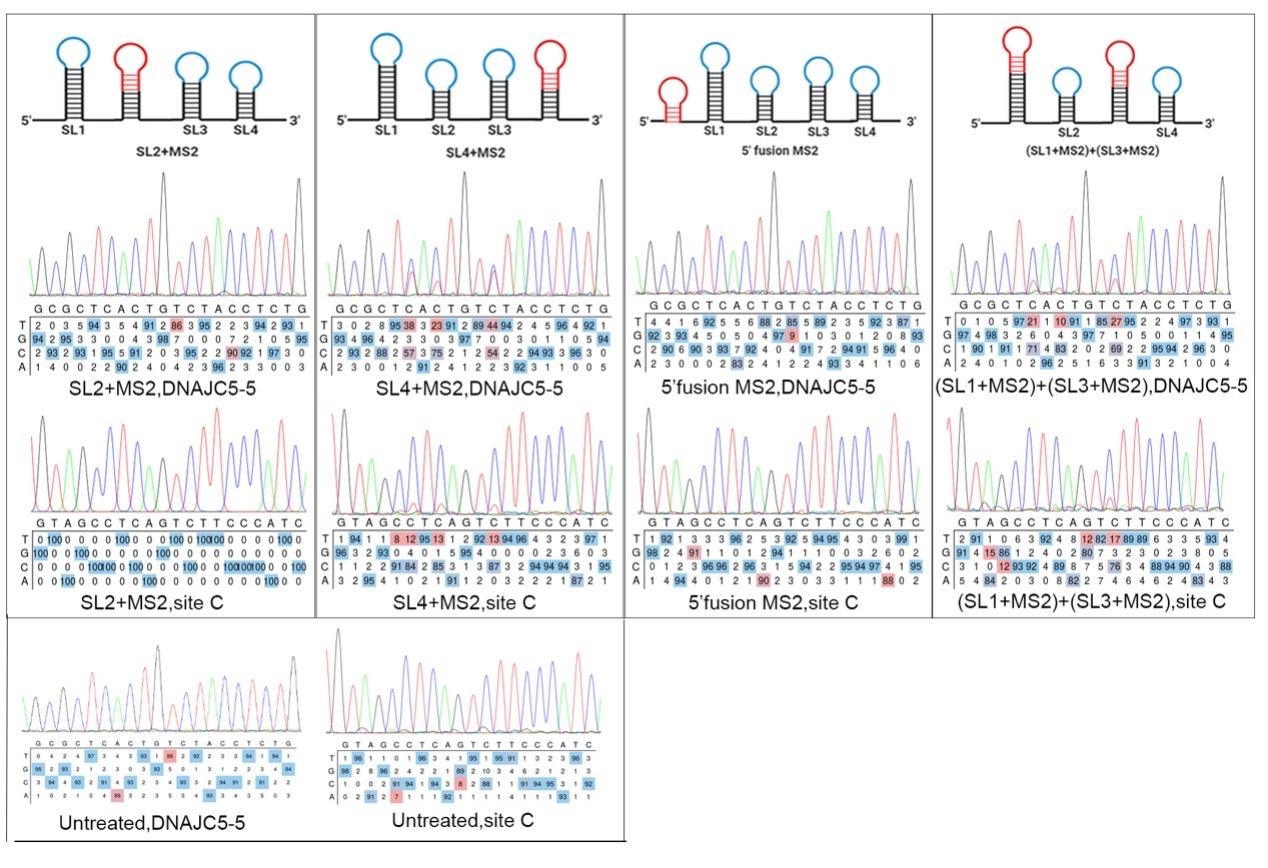

**Figure S2. Representative Sanger sequencing results and EditR analysis for Figure 1d.**


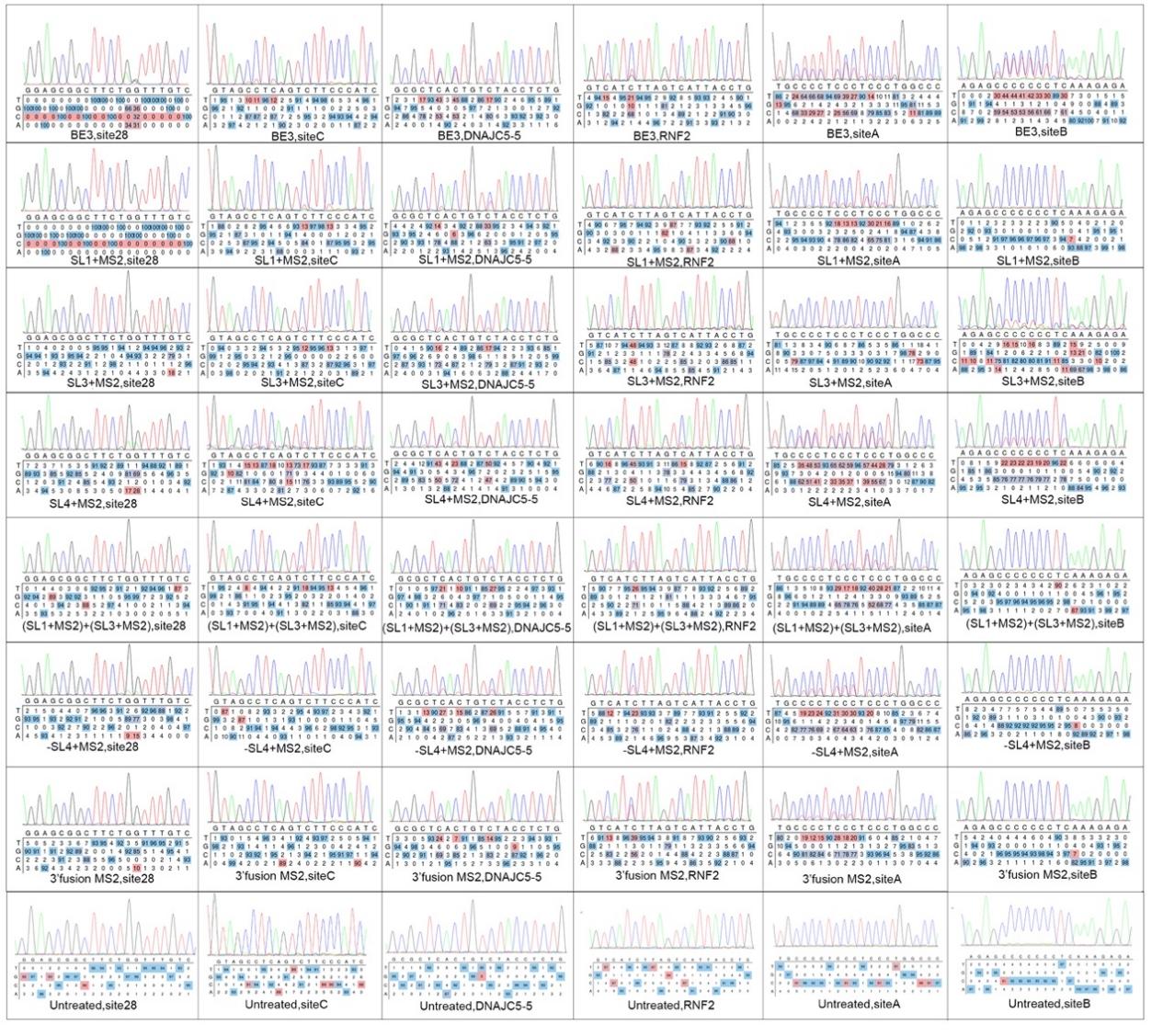
**Figure S3**. **Representative Sanger sequencing results and EditR analysis for Figure 2d.**

**
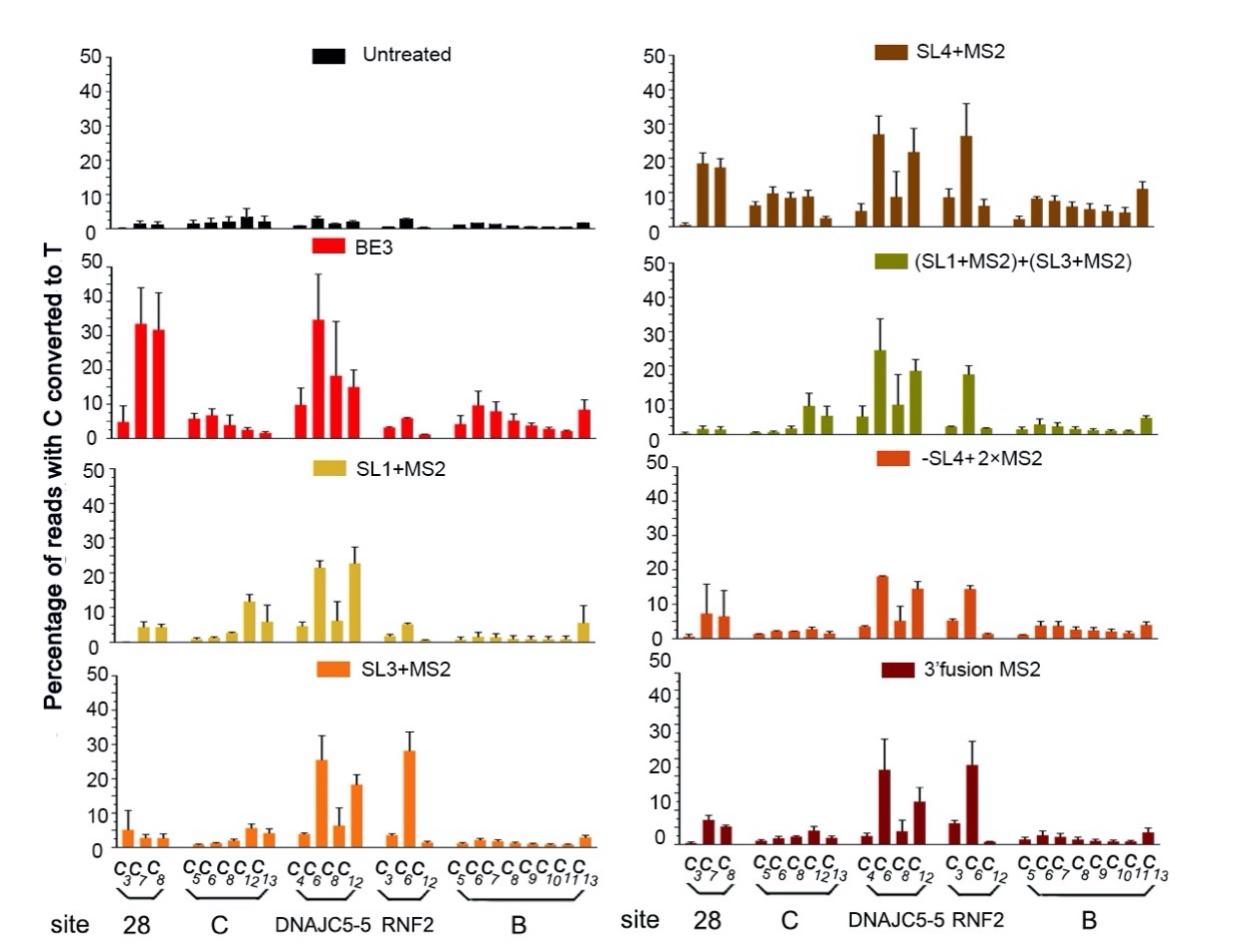
Figure S4. Base editing activity of sgCBEs determined by HTS**. HEK293T cells were transfected with various MS2 sgCBEs targeting a set of five different sites. All the sites were amplified by PCR using primers flanked with different barcodes and the PCR products were subjected to HTS for C-to-T editing analysis. Each experiment was repeated three times, data are represented mean ± SEM.

**
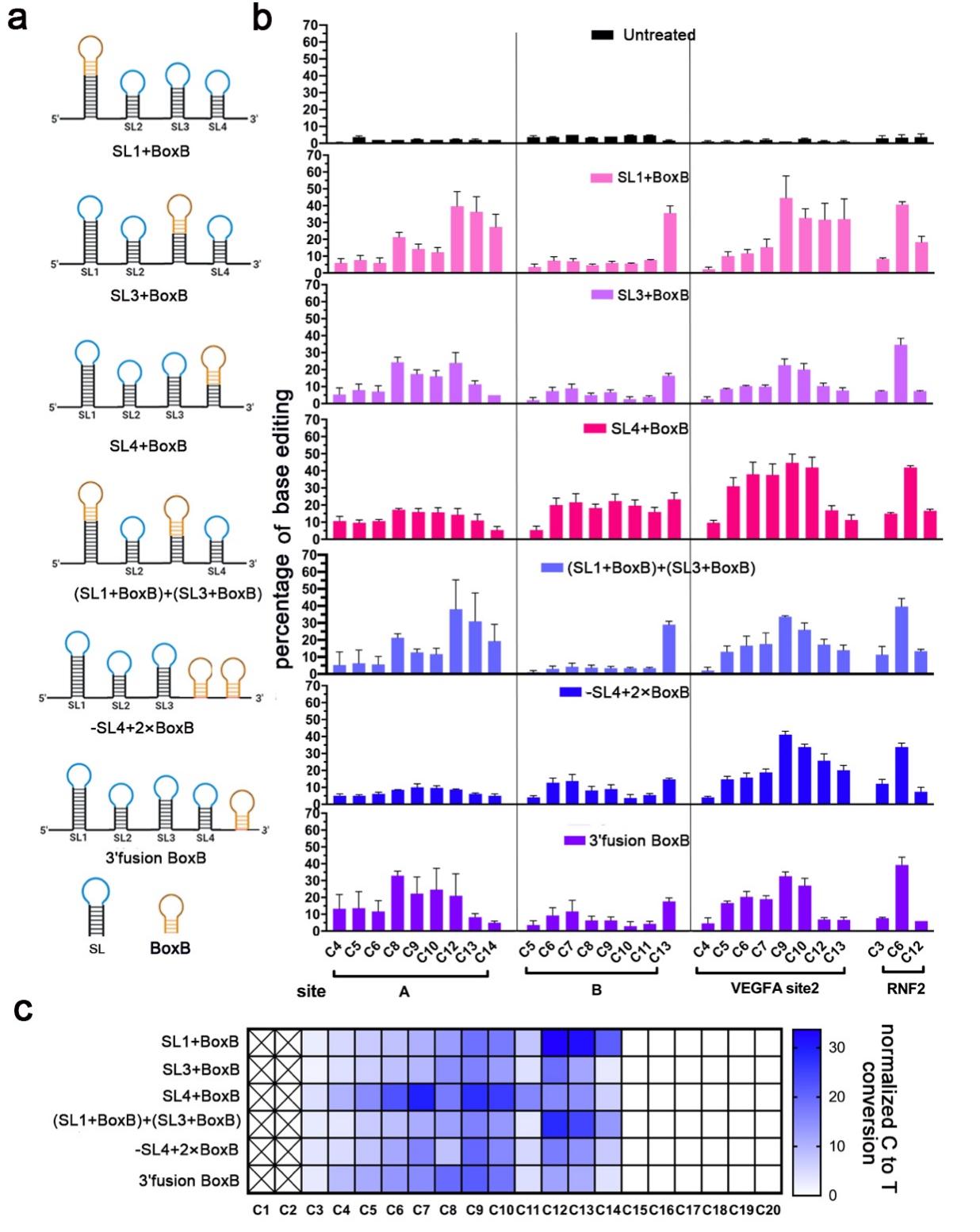
FFigure S5. Base editing of BoxB modified sgCBEs**. (**a)** Organization of BoxB modified sgRNAs (BoxB-sgRNAs). SpCas9 sgRNAs were modified with BoxB tag in a manner similar to MS2-sgRNAs. Sequences of each BoxB modified sgRNAs were shown in Supplementary Note1. **(b)** Cytosine editing of various BoxB-sgRNA derived base editors. HEK293T cells were transfected with plasmids expressing BoxB modified sgCBEs that targeted a set of four different sites. C-to-T editing efficiencies were analyzed by Sanger sequencing and EditR calculating. Each experiment was repeated at least three times, data are represented mean±SEM. **(c)** Heat map showing the base editing window of various BoxB base editors. site A, siteB, VEGFA site2 were used to summarize the editing window and the average C-to-T conversion rate of indicated potion was calculated. Each experiment was repeated three times, data are represented mean ± SEM.


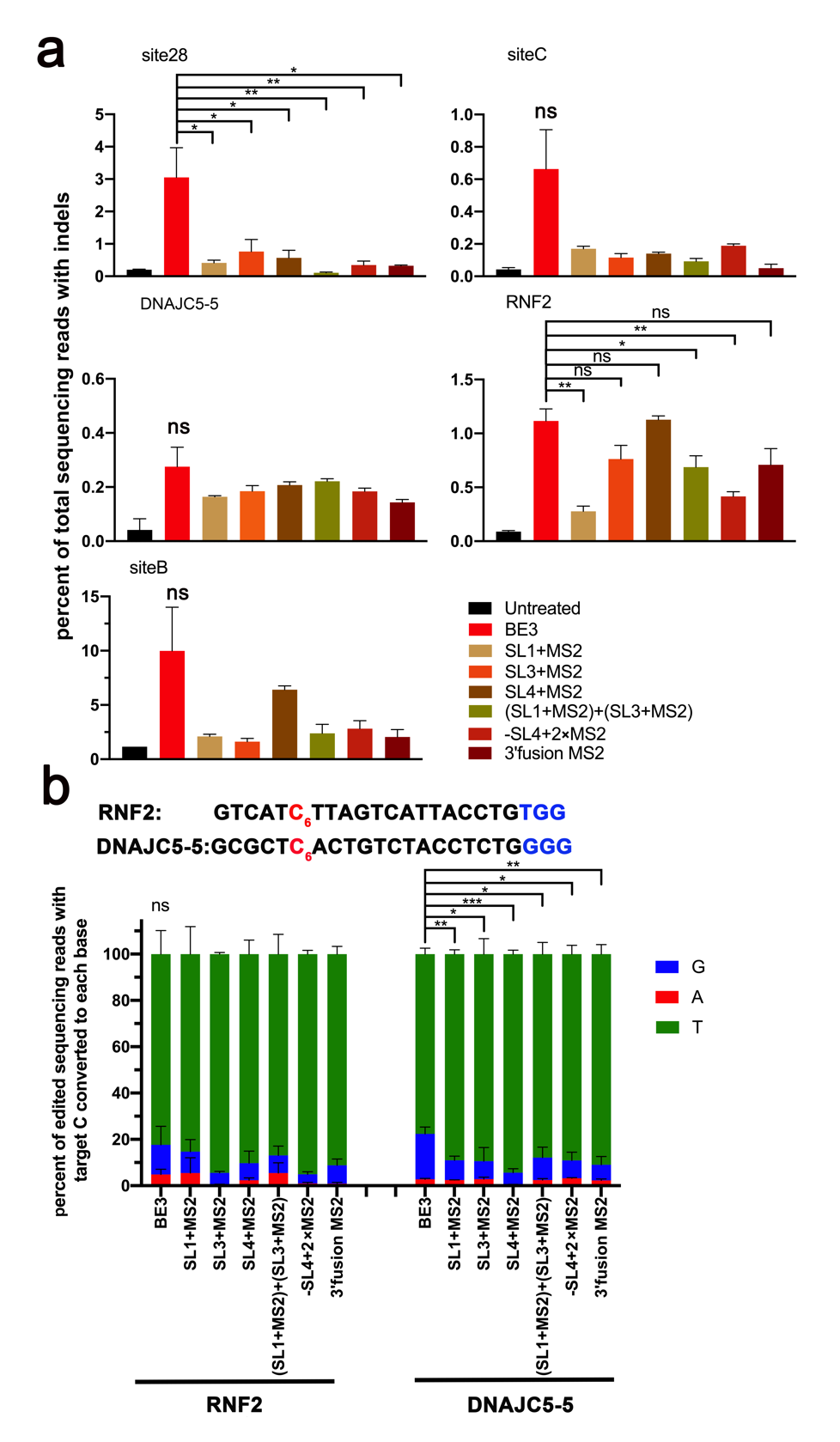


**Figure S6. Indel frequency and product purity of MS2 sgCBEs.** Five targets were subjected to analyze Indel frequency **(a)** and product purity **(b)** of MS2 sgCBEs and BE3 by HTS. Protospacers and PAM (blue) sequences of genomic loci studied, the target Cs in (b) shown in red.For analyzing product purity, two sites harboring obvious level of C-to-Y conversion were analyzed. Each experiment was repeated three times, data are represented mean ± SEM; ns P>0.05, *p<0.05, **p<0.01, ***p<0.001.


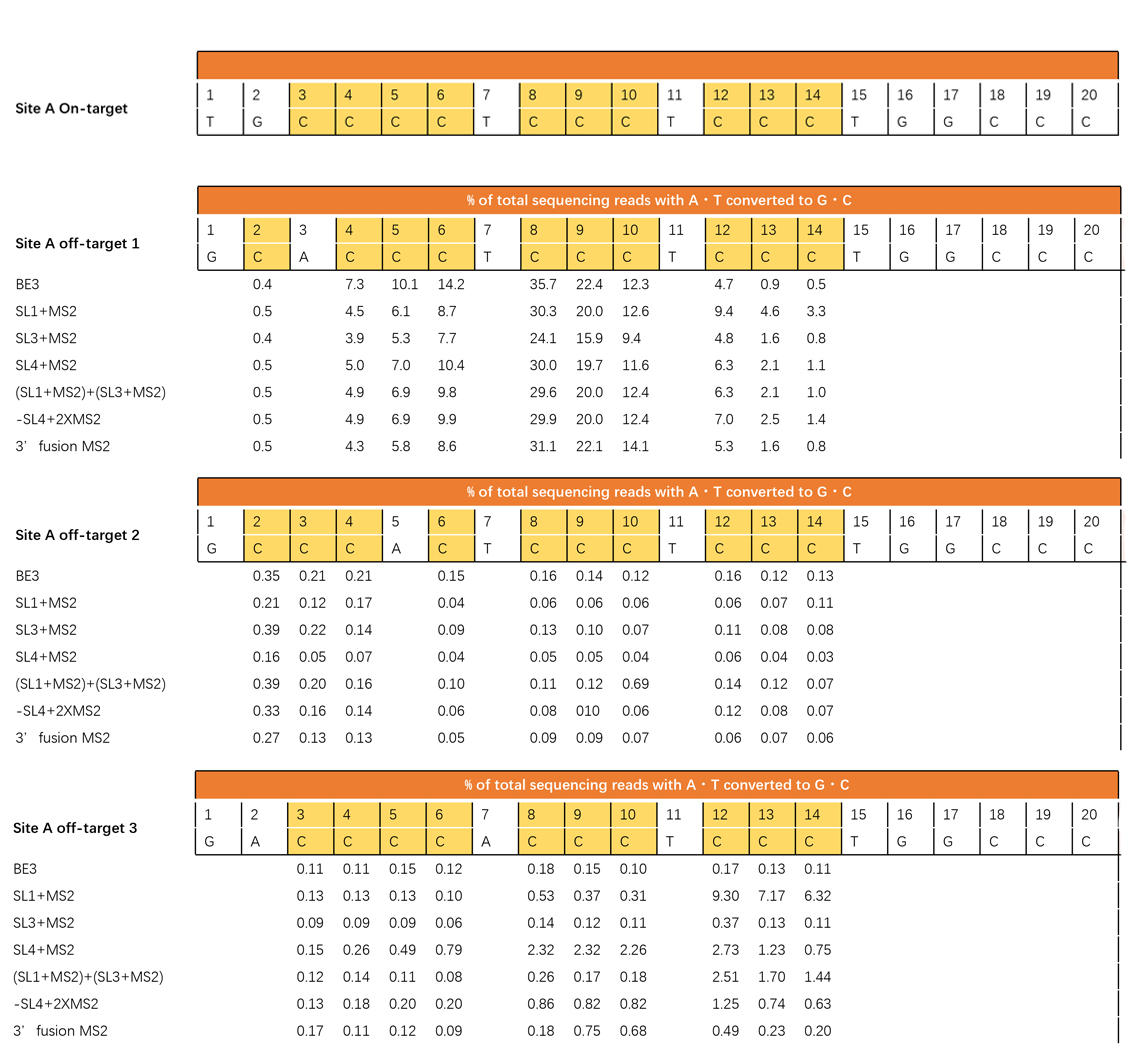


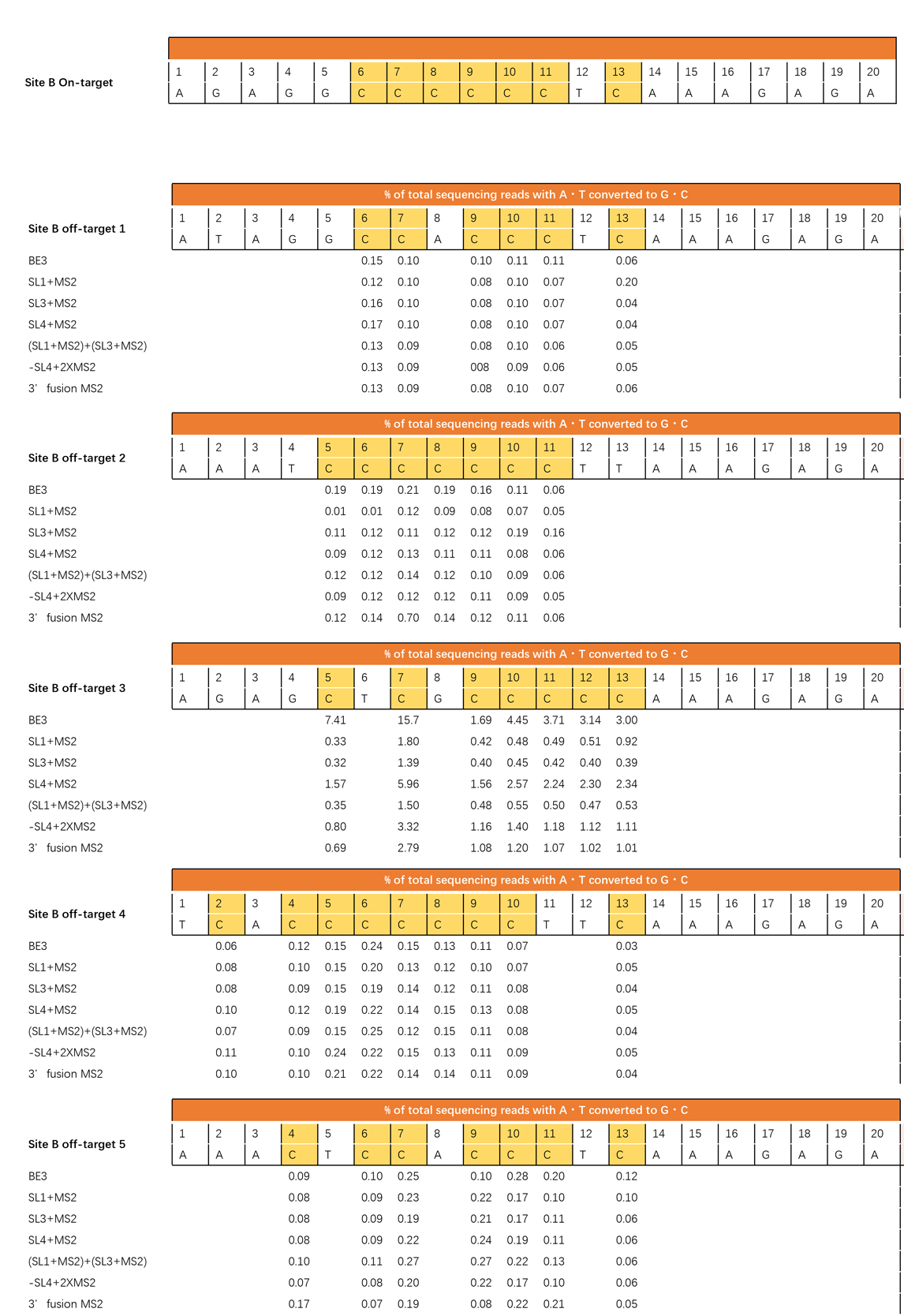


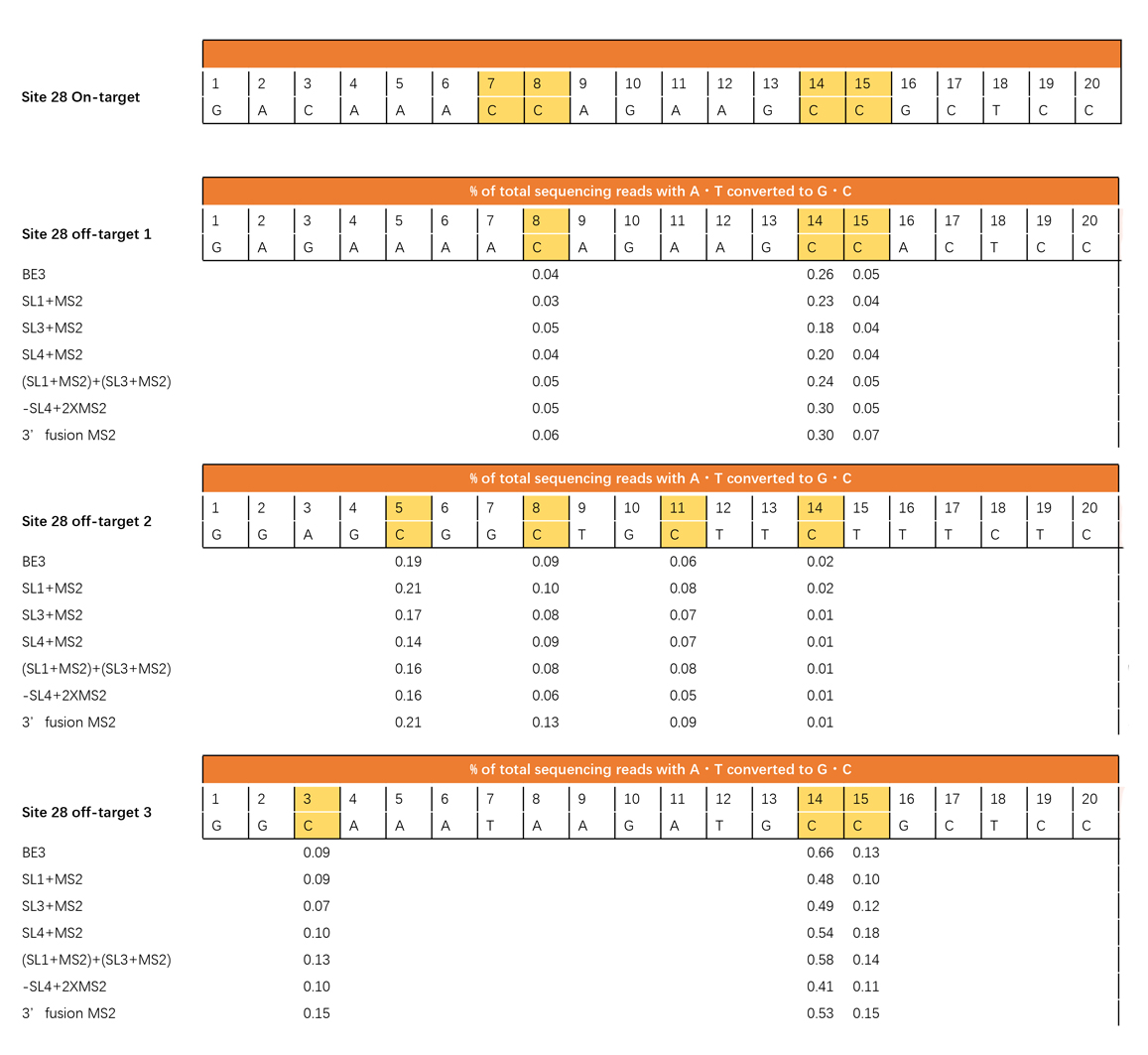

**Figure S7. Sequence dependent DNA off-target editing of sgCBEs and BE3**. Three on-target sites were selected for sequence dependent off-target analysis. The off-target sites were predicted with an on-line program (http://crispr.mit.edu/). All the sites were amplified by PCR using primers flanked with different barcodes and the PCR products were subjected to HTS.


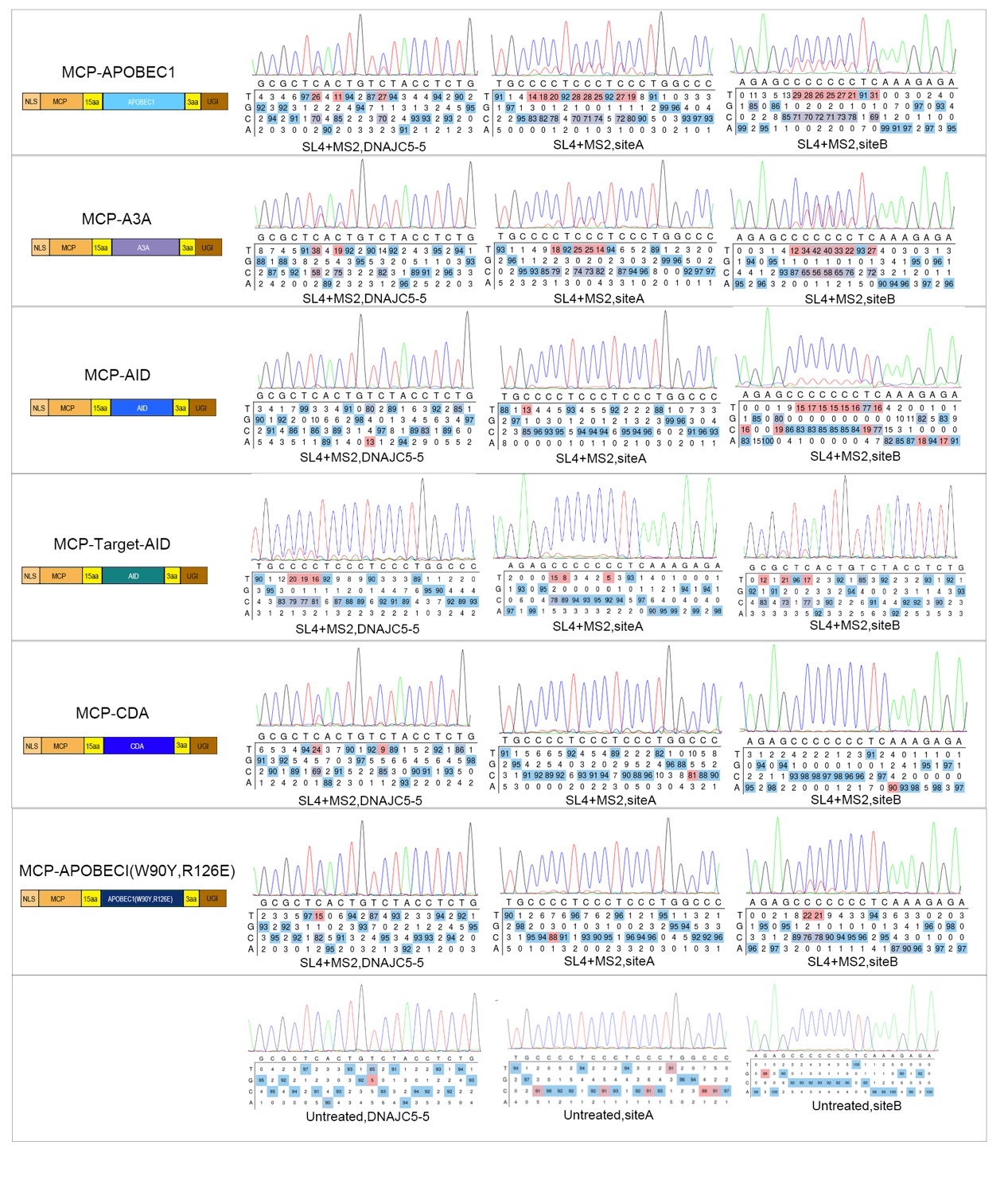


**Figure S8. Representative Sanger sequencing results and EditR analysis for Figure 3b.**


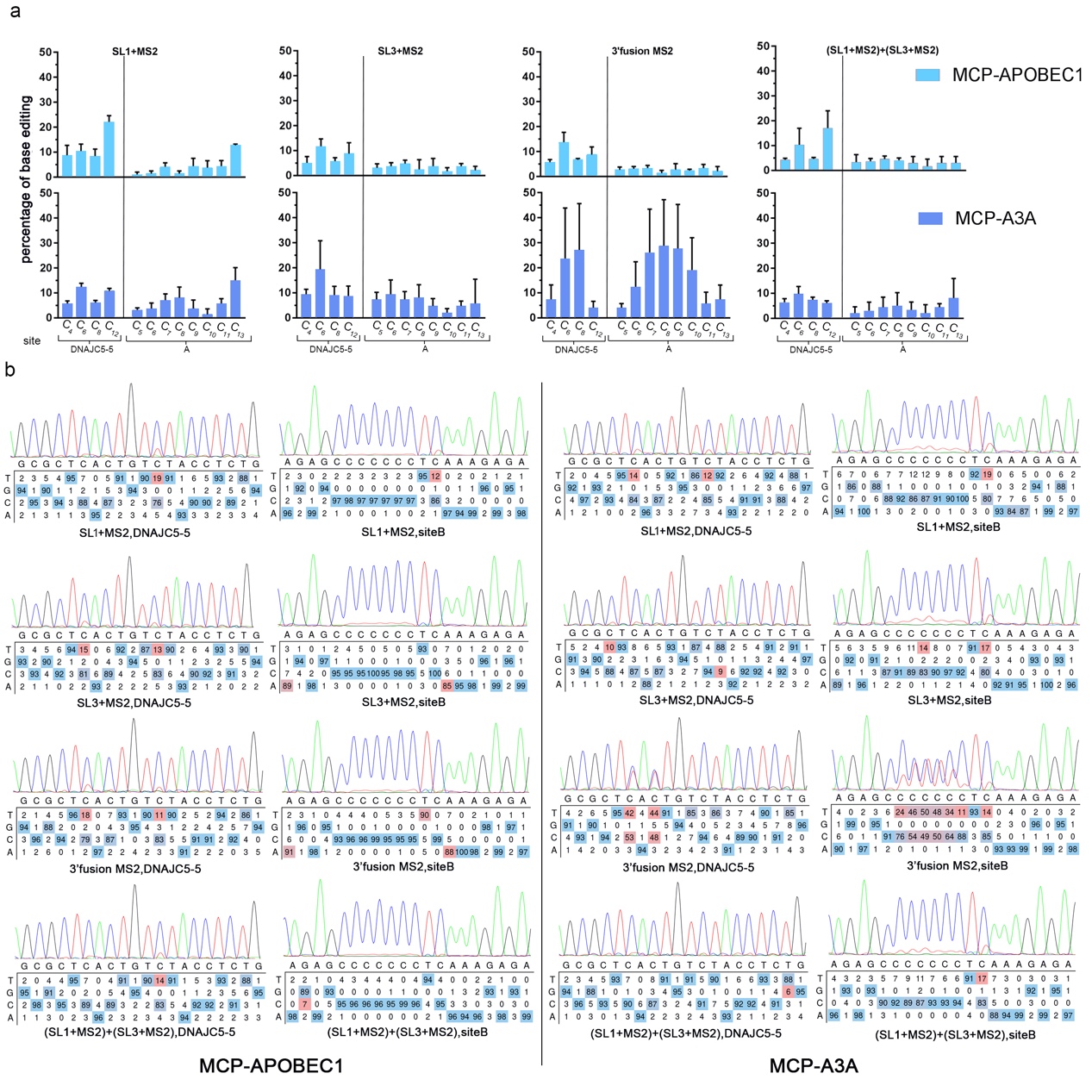


**Figure S9. Comparison between APOBEC1 and A3A derived sg-CBEs in MS2-sgRNA settings other than SL4**. (**a)** Quantitative analysis of C to T editing efficiency of APOBEC1 and A3A derived sg-CBEs in indicated MS2-sgRNAs. HEK293T cells were transfected with plasmids expressing APOBEC1 or A3A derived sg-CBEs targeting a set of two different sites. C-to-T editing efficiencies were analyzed by Sanger sequencing and EditR calculating. C-to-T editing efficiencies were analyzed by Sanger sequencing and EditR calculating. Each experiment was repeated at least three times, data are represented mean±SEM. **(b)** Representative Sanger sequencing results and EditR analysis for Supplementary Figure 9a.


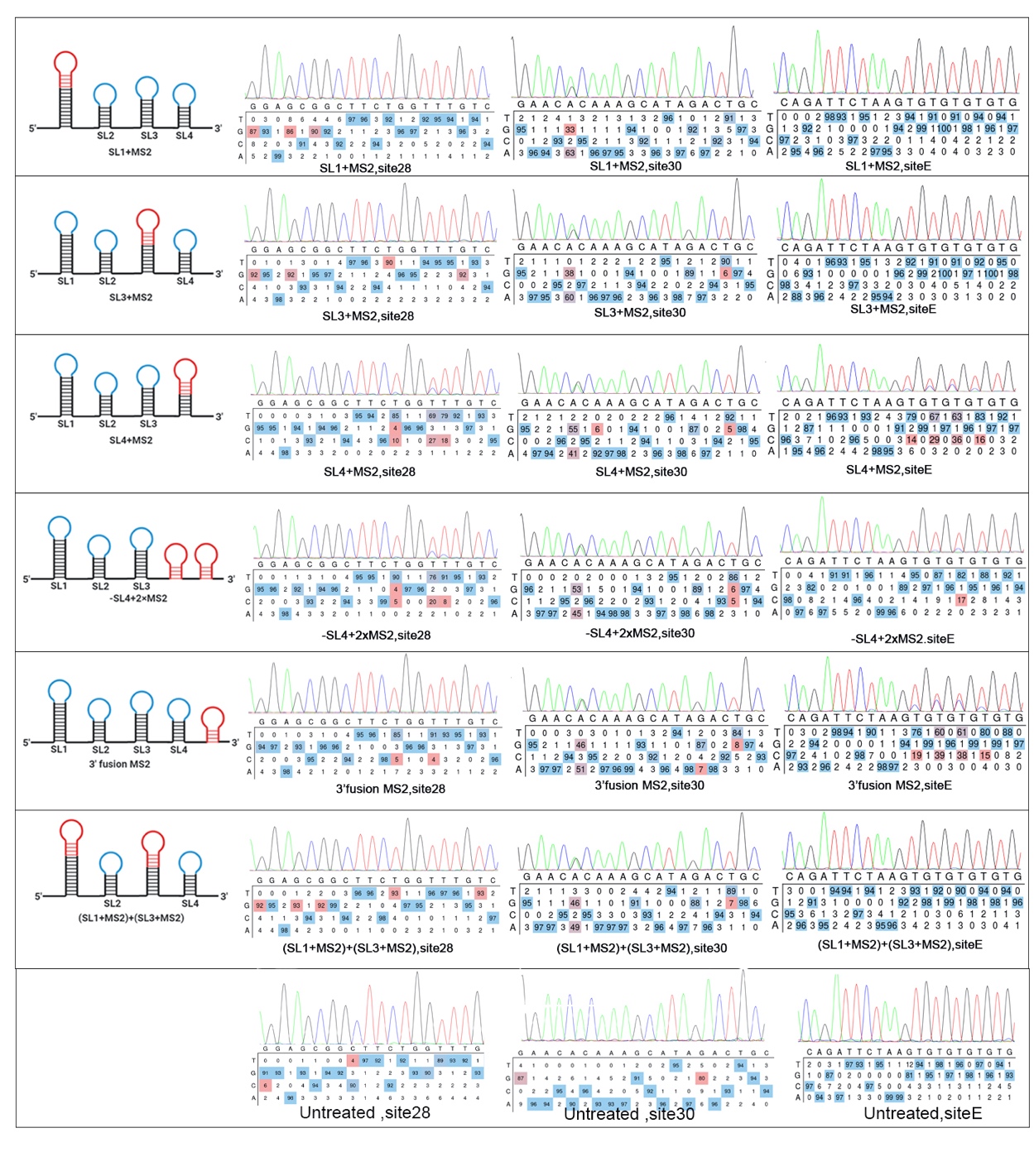


**Figure S10. Representative Sanger sequencing results and EditR analysis for Figure 4b.**


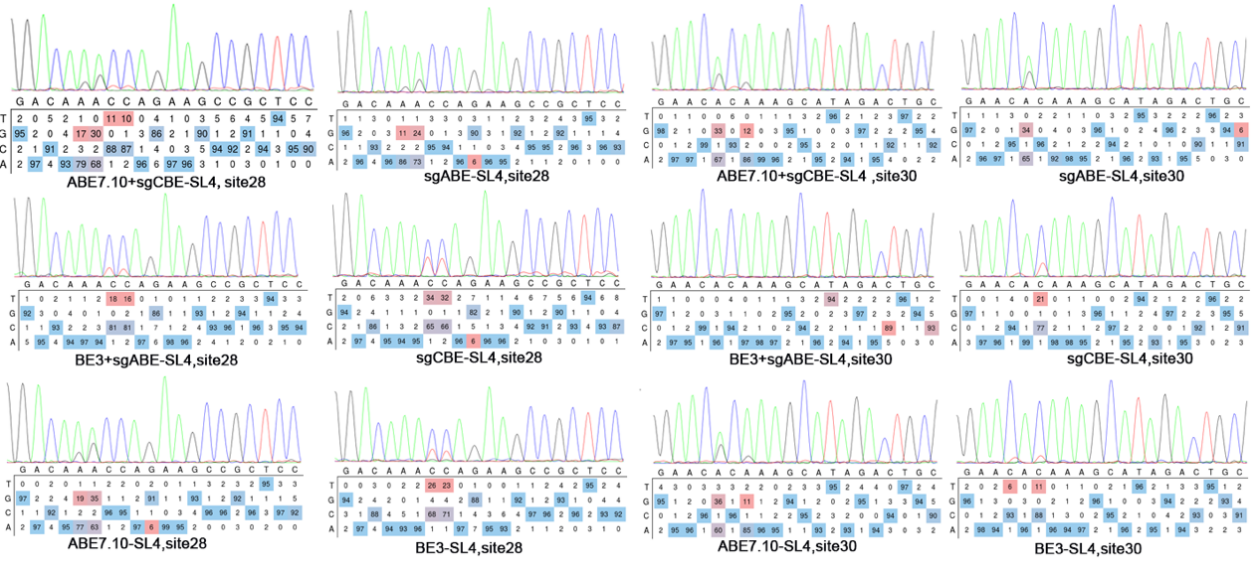

**Figure S11. Representative Sanger sequencing results and EditR analysis for Figure 4d.**

**
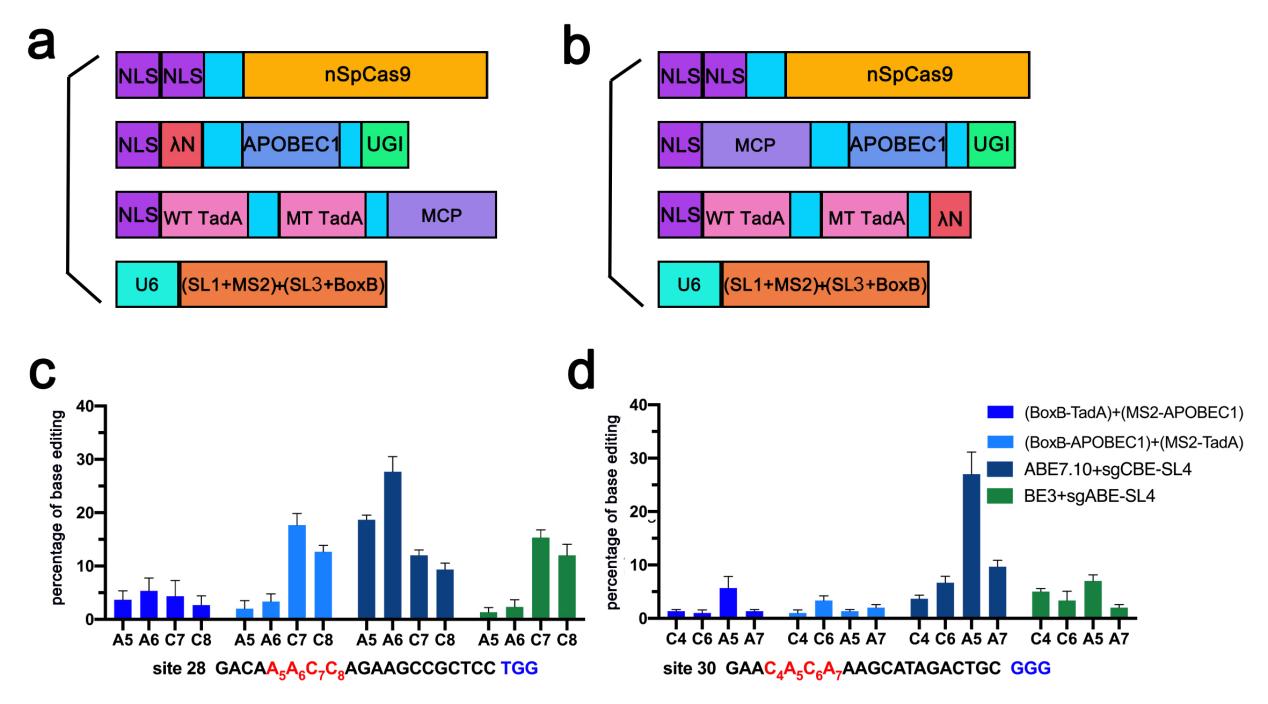
**
**Figure S12. Cytosine and adenosine base editing of dual base editors.** **(a)** and **(b),** organization of sgRNA derived cytosine base editor (sgABE + sgCBE). SpyCas9 D10A nickase, APOBEC1, TadA and MS2+BoxB-sgRNA were expressed separately. **(c)** and **(d)**, base editing of dual tag sgBEs. Plasmids expressing indicated base editors or their combinations were transfected into HEK293T cells. C-to-T and A-to-G editing efficiencies were analyzed by Sanger sequencing and EditR calculating. Targeted cytosines and adenosines were shown in red. C-to-T and A-to-G editing efficiencies were analyzed by Sanger sequencing and EditR calculating. Each experiment was repeated at least three times, data are represented mean ± SEM.


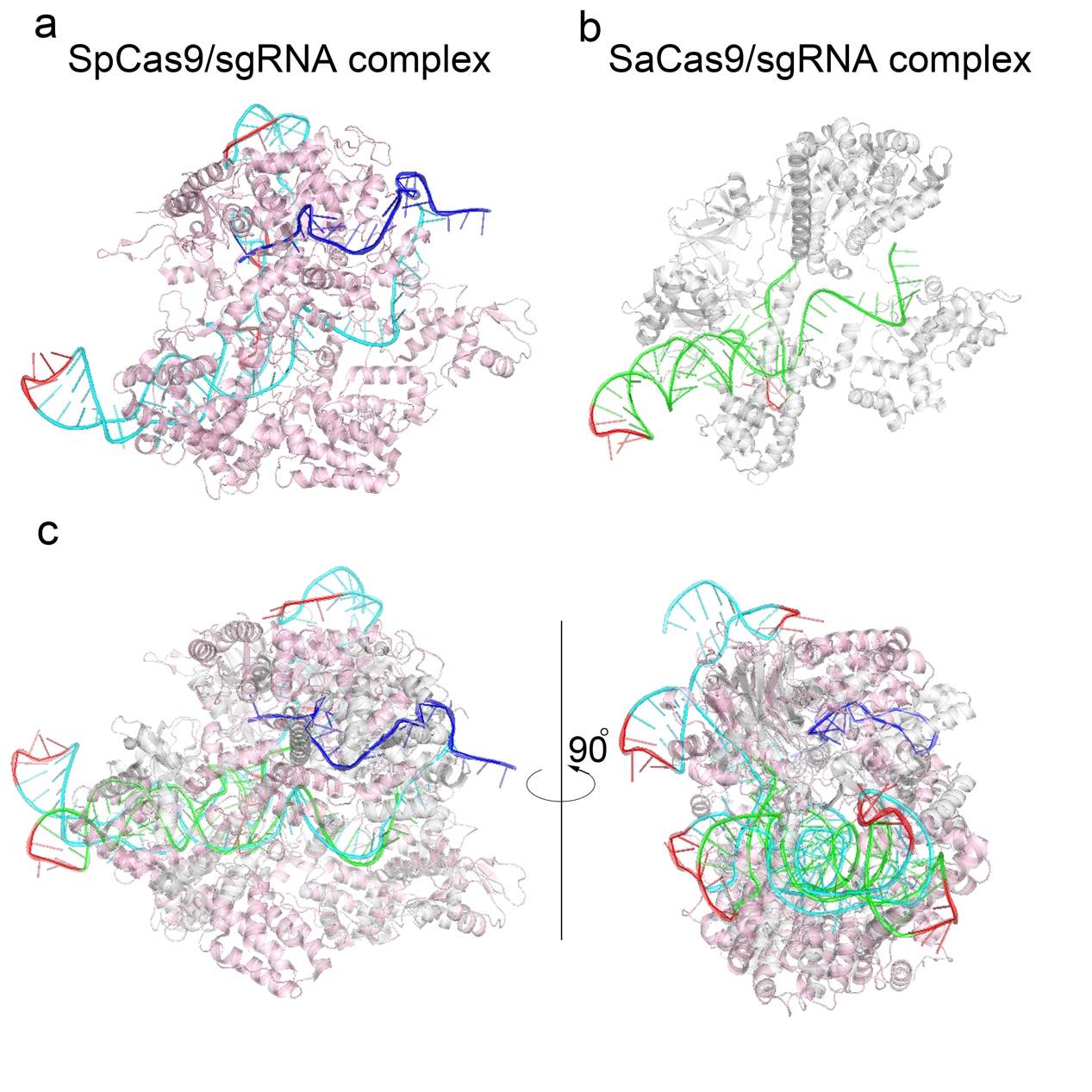


**Figure S13. Comparison between Sa- and Sp-Cas9 complex. (a)** Cartoon of Spcas9/sgRNA/DNA complex. The cyan + green + red parts represented SpCas9 sgRNA scaffold. The blue part represented target strand, and the gray part represented SpCas9. **(b)** Cartoon of SaCas9/RNA complex. the orange + red parts represented SaCas9 sgRNA scaffold, and the purple part represented SaCas9. **(c)** Comparison between Sa-Cas9 and Sp-Cas9 complex. Note that both SpCas9 and SaCas9 were set to be 50% transparency in these cartoons.


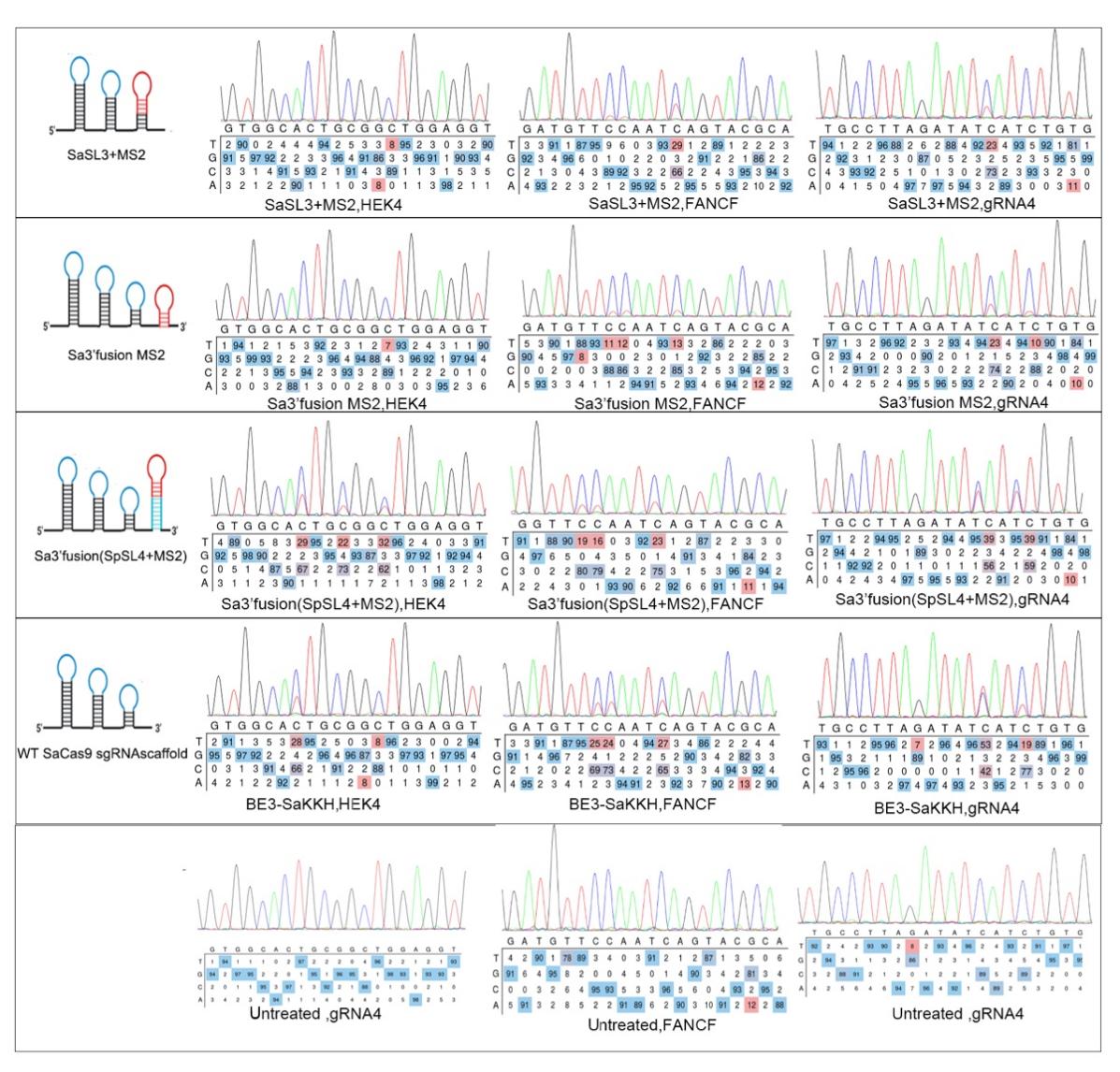


**Figure S14. Representative Sanger sequencing results and EditR analysis for Figure 5c.**

**
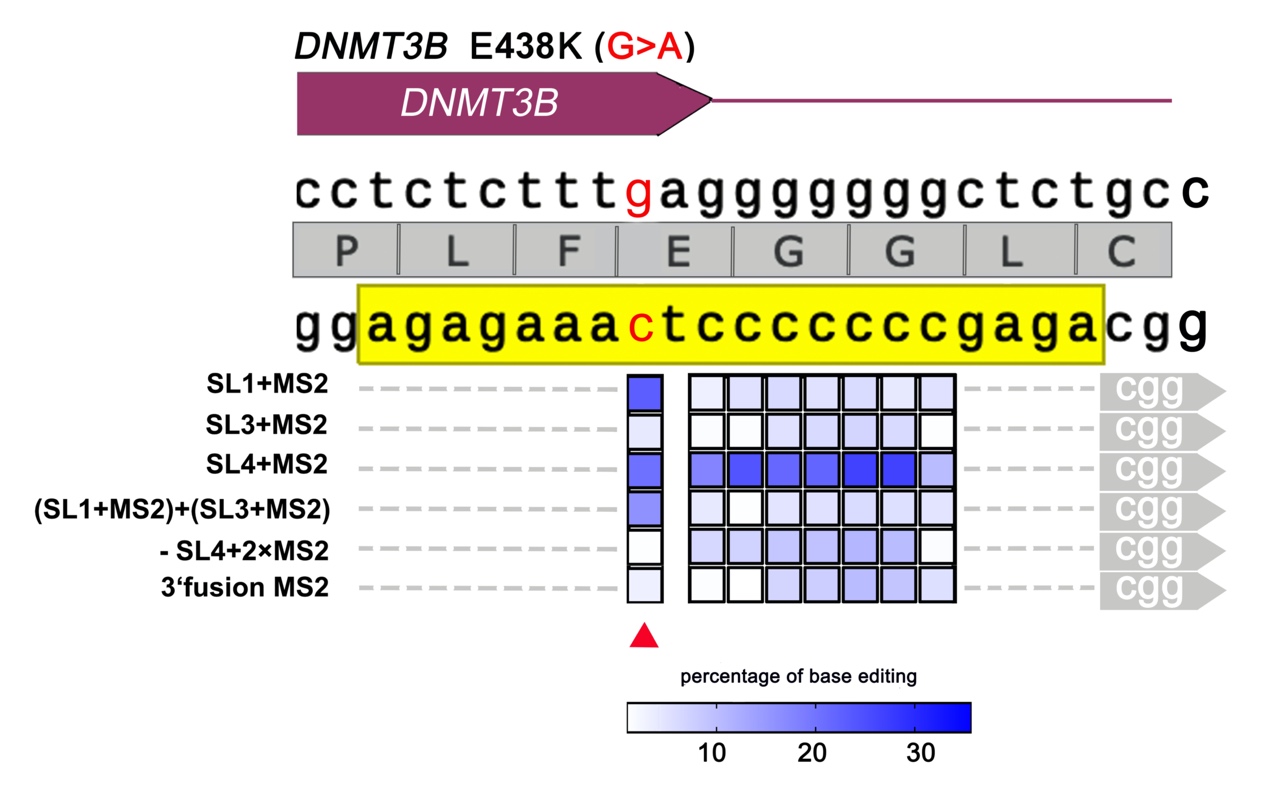
**

**Figure S15. Illustration of the performance of sgCBEs on complicated target.**

E438 of *DNMT3B* was designed as the target. Specificity of each sgCBE in converting E438 to K438 was analyzed. Red triangle indicated the aimed cytosine.

| sgRNA | Target sequence | Oligo-F | Oligo-R | Reference |
| --- | --- | --- | --- | --- |
| DNAJC5-5 | GCGCTCACTGTCTACCTCTG **GGG** | CACCGCGCTCACTGTCTACCTCTG | AAACCAGAGGTAGACAGTGAGCGC | [1] |
| RNF2 | GTCATCTTAGTCATTACCTG **AGG** | CACCGTCATCTTAGTCATTACCTG | AAACCAGGTAATGACTAAGATGAC | [2] |
| site C | GTAGCCTCAGTCTTCCCATC **AGG** | CACCGTAGCCTCAGTCTTCCCATC | AAACGATGGGAAGACTGAGGCTAC | [3] |
| Site 28 | GACAAACCAGAAGCCGCTCC **TGG** | CACCGACAAACCAGAAGCCGCTCC | AAACGGAGCGGCTTCTGGTTTGTC | [4] |
| site A | TGCCCCTCCCTCCCTGGCCC **AGG** | CACCGTGCCCCTCCCTCCCTGGCCC | AAACGGGCCAGGGAGGGAGGGGCAC | [5] |
| site B | AGAGCCCCCCCTCAAAGAGA **GGG** | CACCGAGAGCCCCCCCTCAAAGAGA | AAACTCTCTTTGAGGGGGGGCTCTC | [5] |
| site 30 | GAACACAAAGCATAGACTGC **GGG** | CACCGAACACAAAGCATAGACTGC | AAACGCAGTCTATGCTTTGTGTTC | [4] |
| HEK4 | GTGGCACTGCGGCTGGAGGT **GGGGGT** | CACCGTGGCACTGCGGCTGGAGGT | AAACACCTCCAGCCGCAGTGCCAC | [2] |
| FANCF | GATGTTCCAATCAGTACGCA **GAGAGT** | CACCGATGTTCCAATCAGTACGCA | AAACTGCGTACTGATTGGAACATC | [2] |
| gRNA4 | TGCCTTAGATATCATCTGTG **GAGAAT** | CACCGTGCCTTAGATATCATCTGTG | AAACCACAGATGATATCTAAGGCAC | This study |
| site E | CACACACACACTTAGAATCTG **TGG** | CACCGCACACACACACTTAGAATCTG | AAACCAGATTCTAAGTGTGTGTGTGC | [6] |
| site 32 | GAAGACCAAGGATAGACTGC **TGG** | CACCGAAGACCAAGGATAGACTGC | AAACGCAGTCTATCCTTGGTCTTC | [4] |
| VEGFA site2 | GACCCCCTCCACCCCGCCTC **CGG** | CACCGACCCCCTCCACCCCGCCTC | AAACGAGGCGGGGTGGAGGGGGTC | [7] |
| SHANK3 | GAGCCCTCCCCGACCCACCG **CGG** | CACCGAGCCCTCCCCGACCCACCG | AAACCGGTGGGTCGGGGAGGGCTC | [8] |
| DNMTB site5 | TGTCCCCCATCCTGCCCCAG **AGG** | CACCGTGTCCCCCATCCTGCCCCAG | AAACCTGGGGCAGGATGGGGGACAC | [9] |
| site8-1 | TGCTTCTCCAGCCCTGGCCT **GGG** | CACCGTGCTTCTCCAGCCCTGGCCT | AAACAGGCCAGGGCTGGAGAAGCAC | This study |
| FANCF2 | GGAATCCCTTCTGCAGCACC **TGG** | CACCGGAATCCCTTCTGCAGCACC | AAACGGTGCTGCAGAAGGGATTCC | [10] |
| FANCFsite14-2 | AGAGAACCCAAATCTCCAGG **AGG** | CACCGAGAGAACCCAAATCTCCAGG | AAACCCTGGAGATTTGGGTTCTCTC | This study |
| site9 | GAAGACCAAGGATAGACTGC **TGG** | CACCGAAGACCAAGGATAGACTGC | AAACGCAGTCTATCCTTGGTCTTC | [11] |

**Table S1.** **List of the targets tested in this study**

**Table S2.** **Summary of primers for amplification of each target sites.**

| Target site | chromosome | Forward primer | Reverse primer |
| --- | --- | --- | --- |
| DNAJC5-5 | Chr20 | TCTGTCTGTGCACGTGGCAA | AGCTGTGACCAGTTCAACGC |
| RNF2 | Chr1 | AAACAGTCTTGGTGCC | TCCCTTCCAAATACTAAAATTGTT |
| site C | Chr2 | GCTCTGGACCCTTTATTTGA | CTTGTCCCTCTGTCAATGGC |
| site 28 | Chr3 | GGCACAAAGGATGAAGGCT | GCTCAGTCTTGCATGAAACAC |
| site A | Chr2 | AGAGGAGCTAGGATGCACAG | CCAGCAGCAAGCAGCACTCT |
| site B | Chr20 | GCCTGGAGGGAAATCTTAGG | GGGCTTCACTGAGTCTCCAC |
| site 30 | Chr5 | ACAGGCTACCCCCTAAGT | TCCCAAGTGAGAAGCCAGTG |
| HEK4 | Chr20 | GAACCCAGGTAGCCAGAGAC | TCCTTTCAACCCGAACGGAG |
| FANCF | Chr4 | GGAGACGTTCATGACTGGCA | GGGCCTGGAAGTTCGCTAAT |
| gRNA4 | Chr4 | GAAACAAATGGCGCTCCAGG | CTCTTGTACGGTCCACTTC |
| site E | Chr1 | TTCGAGGTGGAGCTCAAGAT | TTCTGCAGGCGAGAACCTG |
| site32 | Chr22 | TTCCAACCTTCCCACAGG | GGGCATCATAGCGAGAC |
| VEGFA site2 | Chr6 | GAAAGCGACAGGGGCAAAGT | CTCCAATGCACCCAAGACAG |
| SHANK3 | Chr22 | GAGCCCTCCCCGACCCACCG | TCGGGTGACTTCCGCTCCTG |
| DNMTB site5 | Chr20 | GCCTGGAGGGAAATCTTAGG | AGACTGCAGGAACGTAGGAGC |
| site8-1 | Chr9 | TCACAGTGGCAAATGAGGC | CCTGCGTGTAACAAGTGCTC |
| FANCF2 | Chr4 | TTGCCTCCACTGGTTG | CACGGATAAAGACGCTG |
| FANCFsite14-2 | Chr11 | TGCGCTTTACAGGTCTCCAG | ACCTGGTGCAGCAACTCTTT |
| site9 | Chr22 | CAACAGCACTTTCACGCCAC | TCCAAGGATGAGCCTAGGGAT |

**Table S3. HTS Primers used for mammalian cell genomic DNA amplification.**

| sample name | Primer Name | Sequence |
| --- | --- | --- |
| SL1+MS2-siteC | HTS-1-for | gaaggtcGCTCTGTGACCCTTTGTTTGA |
|  | HTS-1-rev | CACAGATGAGAAACTCAGGAG |
| SL3+MS2-siteC | HTS-2-for | gattctgGCTCTGTGACCCTTTGTTTGA |
|  | HTS-2-rev | CACAGATGAGAAACTCAGGAG |
| SL4+MS2-siteC | HTS-3-for | gaacctaGCTCTGTGACCCTTTGTTTGA |
|  | HTS-3-rev | CACAGATGAGAAACTCAGGAG |
| -SL4+2×MS2-siteC | HTS-4-for | gatgacgGCTCTGTGACCCTTTGTTTGA |
|  | HTS-4-rev | CACAGATGAGAAACTCAGGAG |
| 3’fusion-siteC | HTS-5-for | gatgtgcGCTCTGTGACCCTTTGTTTGA |
|  | HTS-5-rev | CACAGATGAGAAACTCAGGAG |
| (SL1+MS2)+(SL3+MS2)-siteC | HTS-6-for | gattgctGCTCTGTGACCCTTTGTTTGA |
|  | HTS-6-rev | CACAGATGAGAAACTCAGGAG |
| BE3-siteC | HTS-7-for | gaactacGCTCTGTGACCCTTTGTTTGA |
|  | HTS-7-rev | CACAGATGAGAAACTCAGGAG |
| Untreated-siteC | HTS-8-for | gaaggtcGCTCTGTGACCCTTTGTTTGA |
|  | HTS-8-rev | CACAGATGAGAAACTCAGGAG |
| SL1-site28 | HTS-9-for | gacgaatGCCCTCTTTTTATTGGAACTGTGG |
|  | HTS-9-rev | CCGACTGGTCCACTTACCTA |
| SL3-site28 | HTS-10-for | gacgttgGCCCTCTTTTTATTGGAACTGTGG |
|  | HTS-10-rev | CCGACTGGTCCACTTACCTA |
| SL4-site28 | HTS-11-for | gaccatcGCCCTCTTTTTATTGGAACTGTGG |
|  | HTS-11-rev | CCGACTGGTCCACTTACCTA |
| -SL4+2×MS2-site28 | HTS-12-for | gagcgatGCCCTCTTTTTATTGGAACTGTGG |
|  | HTS-12-rev | CCGACTGGTCCACTTACCTA |
| 3’fusion-site28 | HTS-13-for | gtatgcaGCCCTCTTTTTATTGGAACTGTGG |
|  | HTS-13-rev | CCGACTGGTCCACTTACCTA |
| (SL1+MS2)+(SL3+MS2)-site28 | HTS-14-for | gagttcgGCCCTCTTTTTATTGGAACTGTGG |
|  | HTS-14-rev | CCGACTGGTCCACTTACCTA |
| BE3-site28 | HTS-15-for | ccgcataGCCCTCTTTTTATTGGAACTGTGG |
|  | HTS-15-rev | CCGACTGGTCCACTTACCTA |
| Untreated-site28 | HTS-16-for | gacgaatGCCCTCTTTTTATTGGAACTGTGG |
|  | HTS-16-rev | CCGACTGGTCCACTTACCTA |
| SL1+MS2-RNF2 | HTS-17-for | gttcgtaGACCATAGCACTTCCCTTCC |
|  | HTS-17-rev | CAAGCATTCCTGACTTCTG |
| SL3+MS2-RNF2 | HTS-18-for | gttgccaGACCATAGCACTTCCCTTCC |
|  | HTS-18-rev | CAAGCATTCCTGACTTCTG |
| SL4+MS2-RNF2 | HTS-19-for | gtacttgGACCATAGCACTTCCCTTCC |
|  | HTS-19-rev | CAAGCATTCCTGACTTCTG |
| -SL4+2×MS2-RNF2 | HTS-20-for | gcattgtGACCATAGCACTTCCCTTCC |
|  | HTS-20-rev | CAAGCATTCCTGACTTCTG |
| 3’fusion MS2-RNF2 | HTS-21-for | gttcaacGACCATAGCACTTCCCTTCC |
|  | HTS-21-rev | CAAGCATTCCTGACTTCTG |
| (SL1+MS2)+(SL3+MS2)-RNF2 | HTS-22-for | gtggtacGACCATAGCACTTCCCTTCC |
|  | HTS-22-rev | CAAGCATTCCTGACTTCTG |
| BE3-RNF2 | HTS-23-for | gctattcGACCATAGCACTTCCCTTCC |
|  | HTS-23-rev | CAAGCATTCCTGACTTCTG |
| Untreated-RNF2 | HTS-24-for | gttcgtaGACCATAGCACTTCCCTTCC |
|  | HTS-24-rev | CAAGCATTCCTGACTTCTG |
| SL1+MS2-DNAJC5-5 | HTS-25-for | gtaaccgCACAAGGCAGTGTTGGATTC |
|  | HTS-25-rev | GACTAAACCACACGGGGCAT |
| SL3+MS2-DNAJC C5-5 | HTS-26-for | gccttagcACAAGGCAGTGTTGGATTC |
|  | HTS-26-rev | GACTAAACCACACGGGGCAT |
| SL4+MS2-DNAJC C5-5 | HTS-27-for | gtaatgccACAAGGCAGTGTTGGATTC |
|  | HTS-27-rev | GACTAAACCACACGGGGCAT |
| -SL4+2×MS2-DNAJC C5-5 | HTS-28-for | ggtatcgcACAAGGCAGTGTTGGATTC |
|  | HTS-28-rev | GACTAAACCACACGGGGCAT |
| 3’fusion MS2-RNF2 | HTS-29-for | ggcctaacACAAGGCAGTGTTGGATTC |
|  | HTS-29-rev | GACTAAACCACACGGGGCAT |
| (SL1+MS2)+(SL3+MS2)-DNAJC5-5 | HTS-30-for | cggtaagcACAAGGCAGTGTTGGATTC |
|  | HTS-30-rev | GACTAAACCACACGGGGCAT |
| BE3-DNAJC5-5 | HTS-31-for | gccaatgcACAAGGCAGTGTTGGATTC |
|  | HTS-31-rev | GACTAAACCACACGGGGCAT |
| Untreated-DNAJC5-5 | HTS-32-for | gtaaccgCACAAGGCAGTGTTGGATTC |
|  | HTS-32-rev | GACTAAACCACACGGGGCAT |
| SL1+MS2-siteB | HTS-33-for | aacctagGTGTCAGGGCCTCAACTG |
|  | HTS-33-rev | GGGCTTCACTGAGTCTCCAC |
| SL3+MS2-siteB | HTS-34-for | aagatgcGTGTCAGGGCCTCAACTG |
|  | HTS-34-rev | GGGCTTCACTGAGTCTCCAC |
| SL4+MS2-siteB | HTS-35-for | aactaggGTGTCAGGGCCTCAACTG |
|  | HTS-35-rev | GGGCTTCACTGAGTCTCCAC |
| -SL4+2×MS2-siteB | HTS-36-for | attctcgGTGTCAGGGCCTCAACTG |
|  | HTS-36-rev | GGGCTTCACTGAGTCTCCAC |
| 3’fusion MS2-siteB | HTS-37-for | attgctcGTGTCAGGGCCTCAACTG |
|  | HTS-37-rev | GGGCTTCACTGAGTCTCCAC |
| (SL1+MS2)+(SL3+MS2)-siteB | HTS-38-for | aagcgtgGTGTCAGGGCCTCAACTG |
|  | HTS-38-rev | GGGCTTCACTGAGTCTCCAC |
| BE3-siteB | HTS-39-for | gccaatgTGTCAGGGCCTCAACTG |
|  | HTS-39-rev | GGGCTTCACTGAGTCTCCAC |
| Untreated-siteB | HTS-40-for | aacctagGTGTCAGGGCCTCAACTG |
|  | HTS-40-rev | GGGCTTCACTGAGTCTCCAC |
| STEME1-siteE-1 | HTS-41-for | gtacagcgcgcatcagCAAGACCTGGCTGAGCTAAC |
|  | HTS-41-rev | AATTGTCCAGCCCCATCTG |
| STEME1-siteE-2 | HTS-42-for | atgtagtagtgagcatCAAGACCTGGCTGAGCTAAC |
|  | HTS-42-rev | AATTGTCCAGCCCCATCTG |
| STEME1-siteE-3 | HTS-43-for | atgtagtagtgagcatCAAGACCTGGCTGAGCTAAC |
|  | HTS-43-rev | AATTGTCCAGCCCCATCTG |
| STEME2-siteE-1 | HTS-44-for | atagacagcgcactgtCAAGACCTGGCTGAGCTAAC |
|  | HTS-44-rev | AATTGTCCAGCCCCATCTG |
| STEME2-siteE-2 | HTS-45-for | acagtacacgagagcaCAAGACCTGGCTGAGCTAAC |
|  | HTS-45-rev | AATTGTCCAGCCCCATCTG |
| STEME2-siteE-3 | HTS-46-for | catgtgtcacactgagCAAGACCTGGCTGAGCTAAC |
|  | HTS-46-rev | AATTGTCCAGCCCCATCTG |
| ABE7.10+sgCBE-SL4-siteE-1 | HTS-47-for | agcgcgtcatcagcatCAAGACCTGGCTGAGCTAAC |
|  | HTS-47-rev | gagcgcagatgctgtcCAAGACCTGGCTGAGCTAAC |
| ABE7.10+sgCBE-SL4-siteE-2 | HTS-48-for | AATTGTCCAGCCCCATCTG |
|  | HTS-48-rev | gagcgcagatgctgtcCAAGACCTGGCTGAGCTAAC |
| ABE7.10+sgCBE-SL4-siteE-3 | HTS-49-for | AATTGTCCAGCCCCATCTG |
|  | HTS-49-rev | ctactgtacgtagtcgCAAGACCTGGCTGAGCTAAC |
| BE3+sgABE-SL4-siteE-1 | HTS-50-for | acagtacacgagagcaCAAGCATTACCTGGGAGCC |
|  | HTS-50-rev | AATTGTCCAGCCCCATCTG |
| BE3+sgABE-SL4-siteE-2 | HTS-51-for | catgtgtcacactgagCAAGCATTACCTGGGAGCC |
|  | HTS-51-rev | CAGGTTCTCGCCTGCAGAAA |
| BE3+sgABE-SL4-siteE-3 | HTS-52-for | agcgcgtcatcagcatCAAGCATTACCTGGGAGCC |
|  | HTS-52-rev | CAGGTTCTCGCCTGCAGAAA |
| Untreated-siteE-1 | HTS-53-for | gagcgcagatgctgtcCAAGCATTACCTGGGAGCC |
|  | HTS-53-rev | CAGGTTCTCGCCTGCAGAAA |
| Untreated-siteE-2 | HTS-54-for | ctactgtacgtagtcgCAAGCATTACCTGGGAGCC |
|  | HTS-54-rev | CAGGTTCTCGCCTGCAGAAA |
| Untreated-siteE-3 | HTS-55-for | gtacagcgcgcatcagCAAGCATTACCTGGGAGCC |
|  | HTS-55-rev | CAGGTTCTCGCCTGCAGAAA |
| STEME1-site9-1 | HTS-56-for | gacatcatgcagtagaTTGGCCCAATGACACCACAT |
|  | HTS-56-rev | AGCTAGAAGGAGAGGCCAGTT |
| STEME1-site9-2 | HTS-57-for | tcagagtcgcacactaTTGGCCCAATGACACCACAT |
|  | HTS-57-rev | AGCTAGAAGGAGAGGCCAGTT |
| STEME1-site9-3 | HTS-58-for | gcatgtcgtgctgataTTGGCCCAATGACACCACAT |
|  | HTS-58-rev | AGCTAGAAGGAGAGGCCAGTT |
| STEME2-site9-1 | HTS-59-for | acgactgctgctcgatTTGGCCCAATGACACCACAT |
|  | HTS-59-rev | AGCTAGAAGGAGAGGCCAGTT |
| STEME2-site9-2 | HTS-60-for | gtgactgcgtgtctagTTGGCCCAATGACACCACAT |
|  | HTS-60-rev | AGCTAGAAGGAGAGGCCAGTT |
| STEME2-site9-3 | HTS-61-for | agagcactactatgcaTTGGCCCAATGACACCACAT |
|  | HTS-61-rev | AGCTAGAAGGAGAGGCCAGTT |
| ABE7.10+sgCBE-SL4-site9-1 | HTS-62-for | gcatgtcgtgctgataTTGGCCCAATGACACCACAT |
|  | HTS-62-rev | AGCTAGAAGGAGAGGCCAGTT |
| ABE7.10+sgCBE-SL4-site9-2 | HTS-63-for | acgactgctgctcgatTTGGCCCAATGACACCACAT |
|  | HTS-63-rev | AGCTAGAAGGAGAGGCCAGTT |
| ABE7.10+sgCBE-SL4-site9-3 | HTS-64-for | gtgactgcgtgtctagTTGGCCCAATGACACCACAT |
|  | HTS-64-rev | AGCTAGAAGGAGAGGCCAGTT |
| BE3+sgABE-SL4-site9-1 | HTS-65-for | agagcactactatgcaTTGGCCCAATGACACCACAT |
|  | HTS-65-rev | AGCTAGAAGGAGAGGCCAGTT |
| BE3+sgABE-SL4-site9-2 | HTS-66-for | atacatagcatcgtagTTGGCCCAATGACACCACAT |
|  | HTS-66-rev | AGCTAGAAGGAGAGGCCAGTT |
| BE3+sgABE-SL4-site9-3 | HTS-67-for | gtcactatatcgtgacTTGGCCCAATGACACCACAT |
|  | HTS-67-rev | AGCTAGAAGGAGAGGCCAGTT |
| Untreated-site9-1 | HTS-68-for | atacatagcatcgtagTTGGCCCAATGACACCACAT |
|  | HTS-68-rev | AGCTAGAAGGAGAGGCCAGTT |
| Untreated-site9-2 | HTS-69-for | gtcactatatcgtgacTTGGCCCAATGACACCACAT |
|  | HTS-69-rev | AGCTAGAAGGAGAGGCCAGTT |
| Untreated-site9-3 | HTS-70-for | gacatcatgcagtagaTTGGCCCAATGACACCACAT |
|  | HTS-70-rev | AGCTAGAAGGAGAGGCCAGTT |
| STEME1-site28-1 | HTS-71-for | tcgtgcatgtgcagtcGCCCTCTTTTTATTGGAACTGTGG |
|  | HTS-71-rev | CCGACTGGTCCACTTACCTA |
| STEME1-site28-2 | HTS-72-for | atatctacgacacgtcGCCCTCTTTTTATTGGAACTGTGG |
|  | HTS-72-rev | CCGACTGGTCCACTTACCTA |
| STEME1-site28-3 | HTS-73-for | ctatagacacagcgatGCCCTCTTTTTATTGGAACTGTGG |
|  | HTS-73-rev | CCGACTGGTCCACTTACCTA |
| STEME2-site28-1 | HTS-74-for | tcgtgcatgtgcagtcGCCCTCTTTTTATTGGAACTGTGG |
|  | HTS-74-rev | CCGACTGGTCCACTTACCTA |
| STEME2-site28-2 | HTS-75-for | atatctacgacacgtcGCCCTCTTTTTATTGGAACTGTGG |
|  | HTS-75-rev | CCGACTGGTCCACTTACCTA |
| STEME2-site28-3 | HTS-76-for | ctatagacacagcgatGCCCTCTTTTTATTGGAACTGTGG |
|  | HTS-76-rev | CCGACTGGTCCACTTACCTA |
| STEME1-site30-1 | HTS-77-for | tagtacactagtcataCAAGACCTGGCTGAGCTAAC |
|  | HTS-77-rev | AATTGTCCAGCCCCATCTG |
| STEME1-site30-2 | HTS-78-for | cgtgagtagtcagacgCAAGACCTGGCTGAGCTAAC |
|  | HTS-78-rev | AATTGTCCAGCCCCATCTG |
| STEME1-site30-3 | HTS-79-for | atcgcatcgcagagacCAAGACCTGGCTGAGCTAAC |
|  | HTS-79-rev | AATTGTCCAGCCCCATCTG |
| STEME2-site30-1 | HTS-80-for | tagtacactagtcataCAAGACCTGGCTGAGCTAAC |
|  | HTS-80-rev | AATTGTCCAGCCCCATCTG |
| STEME2-site30-2 | HTS-81-for | cgtgagtagtcagacgCAAGACCTGGCTGAGCTAAC |
|  | HTS-81-rev | AATTGTCCAGCCCCATCTG |
| STEME2-site30-3 | HTS-82-for | atcgcatcgcagagacCAAGACCTGGCTGAGCTAAC |
|  | HTS-82-rev | AATTGTCCAGCCCCATCTG |

**Table S4. List of the HTS primers used for off-target amplification.**

| Sample name | Forward Primer | Reverse Prime |
| --- | --- | --- |
| SL1+MS2-siteA-off-target1 | CTCAACGagtcggagaccgcaag | ctacagggagtctaagggc |
| SL3+MS2-siteA-off-target1 | CTCGTATagtcggagaccgcaag |  |
| SL4+MS2-siteA-off-target1 | CTCACGAagtcggagaccgcaag |  |
| (SL1+MS2)+(SL3+MS2)-siteA-off-target1 | CCAATGAagtcggagaccgcaag |  |
| -SL4+2×MS2-siteA-off-target1 | CTGCTAAagtcggagaccgcaag |  |
| 3'fusion MS2-siteA-off-target1 | CTGGATGagtcggagaccgcaag |  |
| BE3-siteA-off-target1 | CACACTGagtcggagaccgcaag |  |
| SL1+MS2-siteA-off-target2 | ATTGCTCgtgccagctcttctttacag | cgttatcaccctttcccag |
| SL3+MS2-siteA-off-target2 | ATTCTCGgtgccagctcttctttacag |  |
| SL4+MS2-siteA-off-target2 | CTCAGACgtgccagctcttctttacag |  |
| (SL1+MS2)+(SL3+MS2)-siteA-off-target2 | AACCTAGgtgccagctcttctttacag |  |
| -SL4+2×MS2-siteA-off-target2 | AAGATGCgtgccagctcttctttacag |  |
| 3'fusion MS2-siteA-off-target2 | AAGCGTGgtgccagctcttctttacag |  |
| BE3-siteA-off-target2 | CACTTGAgtgccagctcttctttacag |  |
| SL1+MS2-siteA-off-target3 | CCGTGAAcccaagagcttccagc | gccttcaaagttacccatac |
| SL3+MS2-siteA-off-target3 | CGATTCAcccaagagcttccagc |  |
| SL4+MS2-siteA-off-target3 | CTCTCAGcccaagagcttccagc |  |
| (SL1+MS2)+(SL3+MS2)-siteA-off-target3 | CGTCCATcccaagagcttccagc |  |
| -SL4+2×MS2-siteA-off-target3 | CGTAGAAcccaagagcttccagc |  |
| 3'fusion MS2-siteA-off-target3 | CGTTCGAcccaagagcttccagc |  |
| BE3-siteA-off-target3 | CACTGATcccaagagcttccagc |  |
| SL1+MS2-siteB-off-target1 | AGGTACCttctggcaaagtggctt | ggcagtaccacctgaacac |
| SL3+MS2-siteB-off-target1 | ACCGGTTttctggcaaagtggctt |  |
| SL4+MS2-siteB-off-target1 | CTGTAGA ttctggcaaagtggct |  |
| (SL1+MS2)+(SL3+MS2)-siteB-off-target1 | ATGGCGAttctggcaaagtggctt |  |
| -SL4+2×MS2-siteB-off-target1 | ACTTAGCttctggcaaagtggctt |  |
| 3'fusion MS2-siteB-off-target1 | ACCATCGttctggcaaagtggctt |  |
| BE3-siteB-off-target1 | TACAGAGttctggcaaagtggctt |  |
| SL1+MS2-siteB-off-target2 | ATGCTTC tccctctttattggctacc | ttgagaaatggaaaactcag |
| SL3+MS2-siteB-off-target2 | ATCGTCCtccctctttattggctacc |  |
| SL4+MS2-siteB-off-target2 | GAACACTtccctctttattggctacc |  |
| (SL1+MS2)+(SL3+MS2)-siteB-off-target2 | ATCAAGCtccctctttattggctacc |  |
| -SL4+2×MS2-siteB-off-target2 | ATCCGGAtccctctttattggctacc |  |
| 3'fusion MS2-siteB-off-target2 | ATCGAAGtccctctttattggctac |  |
| BE3-siteB-off-target2 | TAGACGAtccctctttattggctacc |  |
| SL1+MS2-siteB-off-target3 | GTGACTCgccagtttcggcttct | gcttatgcacgacttgtta |
| SL3+MS2-siteB-off-target3 | GTGTCAAgccagtttcggcttct |  |
| SL4+MS2-siteB-off-target3 | GACAGTA gccagtttcggcttct |  |
| (SL1+MS2)+(SL3+MS2)-siteB-off-target3 | AAGAGCTgccagtttcggcttct |  |
| -SL4+2×MS2-siteB-off-target3 | AACGTGTgccagtttcggcttct |  |
| 3'fusion MS2-siteB-off-target3 | AACTGTCgccagtttcggcttct |  |
| BE3-siteB-off-target3 | TTCAGCAgccagtttcggcttct |  |
| SL1+MS2-siteB-off-target4 | GTGACTCcaaatcccatcccgtt | cctgctgtcatccctcta |
| SL3+MS2-siteB-off-target4 | GTGTCAAcaaatcccatcccgtt |  |
| SL4+MS2-siteB-off-target4 | GACAGTAcaaatcccatcccgtt |  |
| (SL1+MS2)+(SL3+MS2)-siteB-off-target4 | AAGAGCTcaaatcccatcccgtt |  |
| -SL4+2×MS2-siteB-off-target4 | AACGTGTcaaatcccatcccgtt |  |
| 3'fusion MS2-siteB-off-target4 | AACTGTCcaaatcccatcccgtt |  |
| BE3-siteB-off-target4 | AGGACTTcaaatcccatcccgtt |  |
| SL1+MS2-siteB-off-target5 | TTCCGATtcagcagccccagataaa | ttgaagtcaccatgcacagc |
| SL3+MS2-siteB-off-target5 | TTCGCAAtcagcagccccagataaa |  |
| SL4+MS2-siteB-off-target5 | GACTCACtcagcagccccagataaa |  |
| (SL1+MS2)+(SL3+MS2)-siteB-off-target5 | TTGCCTAtcagcagccccagataaa |  |
| -SL4+2×MS2-siteB-off-target5 | TTGATCCtcagcagccccagataaa |  |
| 3'fusion MS2-siteB-off-target5 | TTGCAGCtcagcagccccagataaa |  |
| BE3-siteB-off-target5 | TCAACGCtcagcagccccagataaa |  |
| SL1+MS2-site28-off-target1 | ACTTGCaggtagaaagggatgggt | cctgaggacaatggaggt |
| SL3+MS2-site28-off-target1 | ACTCAAGaggtagaaagggatgggt |  |
| SL4+MS2-site28-off-target1 | GAGTCGTaggtagaaagggatgggt |  |
| (SL1+MS2)+(SL3+MS2)-site28-off-target1 | ACGTCTCaggtagaaagggatgggt |  |
| -SL4+2×MS2-site28-off-target1 | ACGTTCTaggtagaaagggatgggt |  |
| 3'fusion MS2-site28-off-target1 | ACTGTTGaggtagaaagggatgggt |  |
| BE3-site28-off-target1 | TCACACGaggtagaaagggatgggt |  |
| SL1+MS2-site28-off-target2 | AGATCACctgtcagaggaactgcttaa | cctgggcaacaataccgaa |
| SL3+MS2-site28-off-target2 | AGACATGctgtcagaggaactgcttaa |  |
| SL4+MS2-site28-off-target2 | GTCACATctgtcagaggaactgcttaa |  |
| (SL1+MS2)+(SL3+MS2)-site28-off-target2 | AGTACAGctgtcagaggaactgcttaa |  |
| -SL4+2×MS2-site28-off-target2 | AGAGCTActgtcagaggaactgcttaa |  |
| 3'fusion MS2-site28-off-target2 | AGAGTGCctgtcagaggaactgcttaa |  |
| BE3-site28-off-target2 | TCAGTACctgtcagaggaactgcttaa |  |
| SL1+MS2-site28-off-target3 | AGTCTACacggaggccctaagtcac | ctgcaccttctatttccaat |
| SL3+MS2-site28-off-target3 | AGTCGTTacggaggccctaagtcac |  |
| SL4+MS2-site28-off-target3 | GTCGTGAacggaggccctaagtcac |  |
| (SL1+MS2)+(SL3+MS2)-site28-off-target3 | AGCTCTGacggaggccctaagtcac |  |
| -SL4+2×MS2-site28-off-target3 | AGCATGAacggaggccctaagtcac |  |
| 3'fusion MS2-site28-off-target3 | AGCAACTacggaggccctaagtcac |  |
| BE3-site28-off-target3 | TTGACAGacggaggccctaagtcac |  |

**Table S5. List of the off-target sites.**

| off-target site | Sequence |
| --- | --- |
| siteA-off-target1 | GCACCCTCCCTCCCTGGCCC |
| siteA-off-target2 | GCCCACTCCCTCCCTGGCCC |
| siteA-off-target3 | CACCCCACCCTCCCTGGCCC |
| siteB-off-target1 | ATAGGCCACCCTCAAAGAGA |
| siteB-off-target2 | AAATCCCCCCCTTAAAGAGA |
| siteB- off-target3 | AGAGCTCGCCCCCAAAGAGA |
| siteB-off-target4 | TCACCCCCCCTTCAAAGAGA |
| siteB-off-target5 | AAACTCCACCCTCAAAGAGA |
| site28-off-target1 | GAGAAAACAGAAGCCACTCC |
| site28-off--target2 | GAGAAAGAAGCAGCCGCTCC |
| site28-off-target3 | GGCAAATAAGATGCCGCTCC |

**Table S6. HTS primers used for amplifying off-target sites.**

| Sample name | Forward Primer | Reverse Prime |
| --- | --- | --- |
| SL1+MS2-siteA-off-target1 | CTCAACGagtcggagaccgcaag | ctacagggagtctaagggc |
| SL3+MS2-siteA-off-target1 | CTCGTATagtcggagaccgcaag |  |
| SL4+MS2-siteA-off-target1 | CTCACGAagtcggagaccgcaag |  |
| (SL1+MS2)+(SL3+MS2)-siteA-off-target1 | CCAATGAagtcggagaccgcaag |  |
| -SL4+2×MS2-siteA-off-target1 | CTGCTAAagtcggagaccgcaag |  |
| 3'fusion MS2-siteA-off-target1 | CTGGATGagtcggagaccgcaag |  |
| BE3-siteA-off-target1 | CACACTGagtcggagaccgcaag |  |
| SL1+MS2-siteA-off-target2 | ATTGCTCgtgccagctcttctttacag | cgttatcaccctttcccag |
| SL3+MS2-siteA-off-target2 | ATTCTCGgtgccagctcttctttacag |  |
| SL4+MS2-siteA-off-target2 | CTCAGACgtgccagctcttctttacag |  |
| (SL1+MS2)+(SL3+MS2)-siteA-off-target2 | AACCTAGgtgccagctcttctttacag |  |
| -SL4+2×MS2-siteA-off-target2 | AAGATGCgtgccagctcttctttacag |  |
| 3'fusion MS2-siteA-off-target2 | AAGCGTGgtgccagctcttctttacag |  |
| BE3-siteA-off-target2 | CACTTGAgtgccagctcttctttacag |  |
| SL1+MS2-siteA-off-target3 | CCGTGAAcccaagagcttccagc | gccttcaaagttacccatac |
| SL3+MS2-siteA-off-target3 | CGATTCAcccaagagcttccagc |  |
| SL4+MS2-siteA-off-target3 | CTCTCAGcccaagagcttccagc |  |
| (SL1+MS2)+(SL3+MS2)-siteA-off-target3 | CGTCCATcccaagagcttccagc |  |
| -SL4+2×MS2-siteA-off-target3 | CGTAGAAcccaagagcttccagc |  |
| 3'fusion MS2-siteA-off-target3 | CGTTCGAcccaagagcttccagc |  |
| BE3-siteA-off-target3 | CACTGATcccaagagcttccagc |  |
| SL1+MS2-siteB-off-target1 | AGGTACCttctggcaaagtggctt | ggcagtaccacctgaacac |
| SL3+MS2-siteB-off-target1 | ACCGGTTttctggcaaagtggctt |  |
| SL4+MS2-siteB-off-target1 | CTGTAGA ttctggcaaagtggct |  |
| (SL1+MS2)+(SL3+MS2)-siteB-off-target1 | ATGGCGAttctggcaaagtggctt |  |
| -SL4+2×MS2-siteB-off-target1 | ACTTAGCttctggcaaagtggctt |  |
| 3'fusion MS2-siteB-off-target1 | ACCATCGttctggcaaagtggctt |  |
| BE3-siteB-off-target1 | TACAGAGttctggcaaagtggctt |  |
| SL1+MS2-siteB-off-target2 | ATGCTTC tccctctttattggctacc | ttgagaaatggaaaactcag |
| SL3+MS2-siteB-off-target2 | ATCGTCCtccctctttattggctacc |  |
| SL4+MS2-siteB-off-target2 | GAACACTtccctctttattggctacc |  |
| (SL1+MS2)+(SL3+MS2)-siteB-off-target2 | ATCAAGCtccctctttattggctacc |  |
| -SL4+2×MS2-siteB-off-target2 | ATCCGGAtccctctttattggctacc |  |
| 3'fusion MS2-siteB-off-target2 | ATCGAAGtccctctttattggctac |  |
| BE3-siteB-off-target2 | TAGACGAtccctctttattggctacc |  |
| SL1+MS2-siteB-off-target3 | GTGACTCgccagtttcggcttct | gcttatgcacgacttgtta |
| SL3+MS2-siteB-off-target3 | GTGTCAAgccagtttcggcttct |  |
| SL4+MS2-siteB-off-target3 | GACAGTA gccagtttcggcttct |  |
| (SL1+MS2)+(SL3+MS2)-siteB-off-target3 | AAGAGCTgccagtttcggcttct |  |
| -SL4+2×MS2-siteB-off-target3 | AACGTGTgccagtttcggcttct |  |
| 3'fusion MS2-siteB-off-target3 | AACTGTCgccagtttcggcttct |  |
| BE3-siteB-off-target3 | TTCAGCAgccagtttcggcttct |  |
| SL1+MS2-siteB-off-target4 | GTGACTCcaaatcccatcccgtt | cctgctgtcatccctcta |
| SL3+MS2-siteB-off-target4 | GTGTCAAcaaatcccatcccgtt |  |
| SL4+MS2-siteB-off-target4 | GACAGTAcaaatcccatcccgtt |  |
| (SL1+MS2)+(SL3+MS2)-siteB-off-target4 | AAGAGCTcaaatcccatcccgtt |  |
| -SL4+2×MS2-siteB-off-target4 | AACGTGTcaaatcccatcccgtt |  |
| 3'fusion MS2-siteB-off-target4 | AACTGTCcaaatcccatcccgtt |  |
| BE3-siteB-off-target4 | AGGACTTcaaatcccatcccgtt |  |
| SL1+MS2-siteB-off-target5 | TTCCGATtcagcagccccagataaa | ttgaagtcaccatgcacagc |
| SL3+MS2-siteB-off-target5 | TTCGCAAtcagcagccccagataaa |  |
| SL4+MS2-siteB-off-target5 | GACTCACtcagcagccccagataaa |  |
| (SL1+MS2)+(SL3+MS2)-siteB-off-target5 | TTGCCTAtcagcagccccagataaa |  |
| -SL4+2×MS2-siteB-off-target5 | TTGATCCtcagcagccccagataaa |  |
| 3'fusion MS2-siteB-off-target5 | TTGCAGCtcagcagccccagataaa |  |
| BE3-siteB-off-target5 | TCAACGCtcagcagccccagataaa |  |
| SL1+MS2-site28-off-target1 | ACTTGCaggtagaaagggatgggt | cctgaggacaatggaggt |
| SL3+MS2-site28-off-target1 | ACTCAAGaggtagaaagggatgggt |  |
| SL4+MS2-site28-off-target1 | GAGTCGTaggtagaaagggatgggt |  |
| (SL1+MS2)+(SL3+MS2)-site28-off-target1 | ACGTCTCaggtagaaagggatgggt |  |
| -SL4+2×MS2-site28-off-target1 | ACGTTCTaggtagaaagggatgggt |  |
| 3'fusion MS2-site28-off-target1 | ACTGTTGaggtagaaagggatgggt |  |
| BE3-site28-off-target1 | TCACACGaggtagaaagggatgggt |  |
| SL1+MS2-site28-off-target2 | AGATCACctgtcagaggaactgcttaa | cctgggcaacaataccgaa |
| SL3+MS2-site28-off-target2 | AGACATGctgtcagaggaactgcttaa |  |
| SL4+MS2-site28-off-target2 | GTCACATctgtcagaggaactgcttaa |  |
| (SL1+MS2)+(SL3+MS2)-site28-off-target2 | AGTACAGctgtcagaggaactgcttaa |  |
| -SL4+2×MS2-site28-off-target2 | AGAGCTActgtcagaggaactgcttaa |  |
| 3'fusion MS2-site28-off-target2 | AGAGTGCctgtcagaggaactgcttaa |  |
| BE3-site28-off-target2 | TCAGTACctgtcagaggaactgcttaa |  |
| SL1+MS2-site28-off-target3 | AGTCTACacggaggccctaagtcac | Ctgcaccttctatttccaat |
| SL3+MS2-site28-off-target3 | AGTCGTTacggaggccctaagtcac |  |
| SL4+MS2-site28-off-target3 | GTCGTGAacggaggccctaagtcac |  |
| (SL1+MS2)+(SL3+MS2)-site28-off-target3 | AGCTCTGacggaggccctaagtcac |  |
| -SL4+2×MS2-site28-off-target3 | AGCATGAacggaggccctaagtcac |  |
| 3'fusion MS2-site28-off-target3 | AGCAACTacggaggccctaagtcac |  |
| BE3-site28-off-target3 | TTGACAGacggaggccctaagtcac |  |

**Note S1. Sequences of the scaffold of individual MS2-sgRNAs and BoxB-sgRNAs.**

**5’ fusion MS2**

ggccaacatgaggatcacccatgtctgcagggccggcaccggGTCTTCgaGAAGACctgttttagagctaGAAAtagcaagttaaaataaggctagtccgttatcaacttgaaaaagtggcaccgagtcggtgcTTTTTT
**SL1+MS2**
GTTTTAGAGCTAGGCCAACATGAGGATCACCCATGTCTGCAGGGCCTAGCAAGTTAAAATAAGGCTAGTCCGTTATCACTTGAAAAAGTGGCACCGAGTCGGTGCTTTTTT
**SL2+MS2**gttttagagctaGAAAtagcaagttaaaataaggcctgccaacatgaggatcacccatgtctgcagggccccgttatcaacttgaaaaagtggcaccgagtcggtgcTTTTTT

**SL3+MS2**gttttagagctaGAAAtagcaagttaaaataaggctagtccgttatcaacttggccaacatgaggatcacccatgtctgcagggccaagtggcaccgagtcggtgcTTTTTT
**SL4+MS2**
gttttagagctaGAAAtagcaagttaaaataaggctagtccgttatcaacttgaaaaagtggcaccgaggccaacatgaggatcacccatgtctgcagggcctcggtgcTTTTTT
**(SL1+MS2)+(SL3+MS2)**
gttttagagctaggccaacatgaggatcacccatgtctgcagggcctagcaagttaaaataaggctagtccgttatcaacttggccaacatgaggatcacccatgtctgcagggccaagtggcaccgagtcggtgctttttt

**-SL4+2×MS2**

gttttagagctaGAAAtagcaagttaaaataaggctagtccgttatcaacttgaaaaagtgggccaacatgaggatcacccatgtctgcagggccggccaacatgaggatcacccatgtctgcagggccTTTTTT

**3’ fusion MS2**

gttttagagctaGAAAtagcaagttaaaataaggctagtccgttatcaacttgaaaaagtggcaccgagtcggtgcggccaacatgaggatcacccatgtctgcagggcc TTTTTT
**saSL3+MS2**GTTTTAGTACTCTGGAAACAGAATCTACTAAAACAAGGCAAAATGCCGTGTTTATCTCGTCAACTggccaacatgaggatcacccatgtctgcagggccTGTTGGCGAGAT TTTTT
**SaSL3 fusion MS2**GTTTTAGTACTCTGGAAACAGAATCTACTAAAACAAGGCAAAATGCCGTGTTTATCTCGTCAACTTGTTGGCGAGATgccaacatgaggatcacccatgtctgcagggccTTTTTT
**SaSL3 fusion (SpSL4+MS2)**
GTTTTAGTACTCTGGAAACAGAATCTACTAAAACAAGGCAAAATGCCGTGTTTATCTCGTCAACTTGTTGGCGAGATggcaccgaggccaacatgaggatcacccatgtctgcagggcctcggtgcTTTTTT

**SL1+BoxB**

GTTTTAGAGCTAGGCCAGGCCCTGAAAAAGGGCCCTGCAGGGCCTAGCAAGTTAAAATAAGGCTAGTCCGTTATCAACTTGAAAAAGTGGCACCGAGTCGGTGCTTTTTT

**SL3+BoxB**

GTTTTAGAGCTAGAAATAGCAAGTTAAAATAAGGCTAGTCCGTTATCAACTTGGCCAGGCCCTGAAAAAGGGCCCTGCAGGGCCAAGTGGCACCGAGTCGGTGCTTTTTT

**SL4+BoxB**

GTTTTAGAGCTAGAAATAGCAAGTTAAAATAAGGCTAGTCCGTTATCAACTTGAAAAAGTGGCACCGAGGCCAGGCCCTGAAAAAGGGCCCTGCAGGGCCTCGGTGCTTTTTT
(**SL1+BoxB)+(SL3+BoxB)**

GTTTTAGAGCTAGGCCAGGCCCTGAAAAAGGGCCCTGCAGGGCCTAGCAAGTTAAAATAAGGCTAGTCCGTTATCAACTTGGCCAGGCCCTGAAAAAGGGCCCTGCAGGGCCAAGTGGCACCGAGTCGGTGCTTTTTT

**-SL4+2×BoxB**

GTTTTAGAGCTAGAAATAGCAAGTTAAAATAAGGCTAGTCCGTTATCAACTTGAAAAAGTGGGCCAGGCCCTGAAAAAGGGCCCTGCAGGGCCGGCCAGGCCCTGAAAAAGGGCCCTGCAGGGCCTTTTTT
**3’fusion BoxB**

GTTTTAGAGCTAGAAATAGCAAGTTAAAATAAGGCTAGTCCGTTATCAACTTGAAAAAGTGGCACCGAGTCGGTGCGGCCAGGCCCTGAAAAAGGGCCCTGCAGGGCCTTTTTT

**Reference:**

1. Yao X, Liu X, Zhang Y, Li Y, Zhao C, Yao S, Wei Y: **Gene Therapy of Adult Neuronal Ceroid Lipofuscinoses with CRISPR/Cas9 in Zebrafish.** *Hum Gene Ther* 2017, **28:**588-597.

2. Komor AC, Zhao KT, Packer MS, Gaudelli NM, Waterbury AL, Koblan LW, Kim YB, Badran AH, Liu DR: **Improved base excision repair inhibition and bacteriophage Mu Gam protein yields C:G-to-T:A base editors with higher efficiency and product purity.** *Sci Adv* 2017, **3:**eaao4774.

3. Wang Y, Zhou L, Liu N, Yao S: **BE-PIGS: a base-editing tool with deaminases inlaid into Cas9 PI domain significantly expanded the editing scope.** *Signal Transduct Target Ther* 2019, **4:**36.

4. Huang TP, Zhao KT, Miller SM, Gaudelli NM, Oakes BL, Fellmann C, Savage DF, Liu DR: **Circularly permuted and PAM-modified Cas9 variants broaden the targeting scope of base editors.** *Nat Biotechnol* 2019, **37:**626-631.

5. Kim YB, Komor AC, Levy JM, Packer MS, Zhao KT, Liu DR: **Increasing the genome-targeting scope and precision of base editing with engineered Cas9-cytidine deaminase fusions.** *Nat Biotechnol* 2017, **35:**371-376.

6. Cheng TL, Li S, Yuan B, Wang X, Zhou W, Qiu Z: **Expanding C-T base editing toolkit with diversified cytidine deaminases.** *Nat Commun* 2019, **10:**3612.

7. Liu Y, Li G, Yang G, Gu H, Huang S, Yu W, Qin G, Liu X, Zhou F, Huang X, Wei Y: **Increasing the targeting scope and efficiency of base editing with Proxy-BE strategy.** *FEBS Lett* 2020, **594:**1319-1328.

8. Nishimasu H, Shi X, Ishiguro S, Gao L, Hirano S, Okazaki S, Noda T, Abudayyeh OO, Gootenberg JS, Mori H, et al: **Engineered CRISPR-Cas9 nuclease with expanded targeting space.** *Science* 2018, **361:**1259-1262.

9. Zuo E, Sun Y, Yuan T, He B, Zhou C, Ying W, Liu J, Wei W, Zeng R, Li Y, Yang H: **A rationally engineered cytosine base editor retains high on-target activity while reducing both DNA and RNA off-target effects.** *Nat Methods* 2020, **17:**600-604.

10. Anzalone AV, Randolph PB, Davis JR, Sousa AA, Koblan LW, Levy JM, Chen PJ, Wilson C, Newby GA, Raguram A, Liu DR: **Search-and-replace genome editing without double-strand breaks or donor DNA.** *Nature* 2019, **576:**149-157.

11. Grunewald J, Zhou R, Lareau CA, Garcia SP, Iyer S, Miller BR, Langner LM, Hsu JY, Aryee MJ, Joung JK: **A dual-deaminase CRISPR base editor enables concurrent adenine and cytosine editing.** *Nat Biotechnol* 2020, **38:**861-864.
