# Supplementary material for: sgBE: a structure-guided design of sgRNA architecture specifies base editing window and enables simultaneous conversion of cytosine and adenosine
Source: Genome Biol. 2020 Aug 28;21:222. doi: 10.1186/s13059-020-02137-6 (PMC7453718; doi:10.1186/s13059-020-02137-6)
Supplement: Supplementary file 2 — Additional file 2. Review history. [file 13059_2020_2137_MOESM2_ESM.docx]

Review History

**First round of review**

**Reviewer 1**

**Were you able to assess all statistics in the manuscript, including the appropriateness of statistical tests used?**

No, I do not feel adequately qualified to assess the statistics.

**Comments to author:**

This manuscript presented by Wang and colleagues described a base editing system through tethering of deaminases with modified sgRNA scaffold via MS2/MCP interaction. The authors first tested the optimal position in sgRNA to insert the MS2 sequence which induced highest editing efficiency through co-transfection of MCP-Apobec1/UGI plasmids. Then this system was expanded to adenine deaminase and demonstrated the possibility of simultaneous A/C base conversion with the same target. The system is interesting, but it is a preliminary study for the characterization of this system, more extensive evaluation and application should be performed.

Major concern:

1. Novelty: Since the strategy has been demonstrated in previous report (Nature Methods, 2016), it is easy to speculate that similar strategy would function. Comparing the data, it seems that the activity of sgBE is higher than CRISPR-X, but direct comparison should be done in this study. Moreover, there are different RNA/RBP systems adapted for CRISPR system, a profound study besides MS2/MCP system should be performed to obtain a optimized system.

2. Specificity: Specificity is a critical issue to evaluate the base editing system especially the authors mentioned the potential clinical applications. However, there is no single data to show the sequence dependent or independent off-target effects of the sgBE system. Since this study using a free deaminase overexpression strategy, the sequence independent off-target editing effects should be higher. Analysis of these Cas9 independent off-targets will be critical to demonstrate if these base editors will be therapeutically useful. While whole genome sequencing may be outside the scope of this paper, the authors should at least comment on this potential downside of the technology. However, the sequence dependent off-target effects should be extensively studied. Moreover, RNA off-targeting should be tested to show whether sgBE system increases side effect. Previous study showed AID exhibited very low RNA off-targeting, could the system be optimized and adapted to AID, since the author showed sgBE-AID was not efficient currently.

3. Efficiency: The previous reported CRISPR-X system showed very limited activity. Through test of variant deaminases and using Cas9n instead of dCas9, the seBE system showed much higher activity than CRISPR-X. Thus the increase of efficiency is the shining point, but only a few targets have been investigated making the advantage unconvincing. To analyze the editing efficiency, deep-sequencing analysis should be applied. The EditR program is not accurate for base conversions. Moreover, since BE3 or BE4 and other advanced CBEs have shown very high efficiency and specificity, directly comparing of sgBE with BE3 should be performed for more targets.

4. Products purity and indel rates: The products purity and indel rates of sgBE should be examined.

5. Editing window: The author claimed they were able to diversify the tool-box with a series of pattern-specific editors. It is interesting, but again only few targets have been tested. It is hard to draw any conclusion. Moreover, if it is true, what is the advantage of BEs with wider editing window? The authors should discuss the feature and show the utility.

6. Dual base editors: It is interesting to generate simultaneous A/C mutation, but what is the ratio of single A>G, C>T and simultaneous A/C allele? More targets and deep-sequencing should be performed. Since the authors claimed that (SL1+MS2)+(SL3_MS2) sgRNA showed two peaks, why not using two different scaffolds and RBPs to recruit cytosine and adenine deaminases to achieve simultaneous mutation. The strategy used in this study to create simultaneous A/C mutation seems not a optimal one. Moreover, recent publication by Caixiao Gao's group has shown a new system called STEME to generate simultaneous A/C mutation. It is better to compare these two system.

7. Utility: This study did not show any application, although they claimed that the system could be easily packaged into AAV. However, as I know not single vector can package sgBEs. Since numerous studies have shown efficient editing of traditional BE3/BE4 to treat genetic diseases through intein-mediated AAV system, only discussion is not enough to show the advantage.

8. From the discussion and the results, the advantages as well as the characteristics of sgBEs were not fully presented.

**Reviewer 2**

**Were you able to assess all statistics in the manuscript, including the appropriateness of statistical tests used?**

There are no statistics in the manuscript.

**Comments to author:**

Wang et al develop an approach to recruit cytosine and adenine deaminase catalytic domains to a target site for base editing using sgRNA scaffolds with embedded MS2-targeting domains that bind to a separately co-delivered MS2-deaminase fusion protein. They observe reasonable editing rates and find that incorporating MS2 domains within different sgRNA structural elements can alter the editing window, which is an interesting finding. They show that this approach can enable dual A>G and C>T editing within a single target, though with some caveats related to efficiency.

The authors describe an approach similar to one established Hess et al, though that strategy was primarily focused on driving somatic hypermutation. A prior study also established that adenosine and cytosine deaminase domains could both be fused to a Cas9 scaffold to facilitate dual editing. The authors method is distinct from that approach and could offer the advantage of more flexibility owing to the potential for editing window tuning. This work is interesting and should serve as a valuable editing to the growing base editing toolbox. However, I have some concerns. The authors must expand the number of sites they targeted with some constructs and generate design rules that could help the community capitalize on their findings. Specifically, right now, the results are only observations, and leave much to be desired in terms of forward design and implementation. Additionally, there is insufficient evidence to support some claims, including the application of this approach for AAV.

Major comments:

1. In Fig. 1C, the authors should include the editing frequencies observed using a native cytidine deaminase. These control points appear to be missing from this data and other data throughout the manuscript.

2. On Pg 5, line 9, the authors say, "editing efficiency in each targetable cytosines by SL+MS2 sgRNA was ~40% higher than that by (SL1+MS2)+(SL3+MS2) sgRNA ( 11.55% VS 16.14%) (Fig. 1c and Additional file 1: Fig. S1)." This sentence is unclear to me. Do they mean the inverse? That (SL1+MS2)+(SL3+MS2) sgRNA was higher than SL+MS2 sgRNA? Also, the percentages do not match the data being discussed. A 40% increase was only observed in Fig. 1C left, which doesn't seem to be the data mentioned in this sentence.

3. The authors conclude that SL4-MS2 has a much broader editing window than (SL1+MS2)+(SL3+MS2). I think there is insufficient evidence to fully draw this conclusion. I would like to see the authors target additional sequences and confirm the trend on multiple sites.

4. In Pg. 5, the authors note that different sgCBEs gave unique editing patterns. They should elaborate more on their findings. Additionally, are there any design principles that can be learned from their results?

5. The authors created an SaCas9-based sgCBE construct, which they say demonstrates compatibility with AAV delivery. This is not convincingly established to me. For one, SaCas9 cytidine deaminases still exceed the carrying capacity of a single AAV vector. So it is unclear as to what hurdle the authors are overcoming. Two, they do not demonstrate delivery of the SaCas9-sgCBE by AAV. The authors should at least transduce 293 cells with AAV encoding the system to claim they have developed a translational system.

Minor comments:

1. In Fig. 1, in addition to the 3D structure showed in panel A, a two-dimensional schematic of sgRNA secondary structure with labeling of its specific structures (stem loops) would be useful (similar to that in Fig. 2b).

2. The authors are interested in developing tools compatible with AAV delivery, thus it would be useful to indicate the size (in kb) of the constructs in the figures (for example, Fig. 1b which shows three different constructs).

3. The axises in the figures need to be better labeled. For example, in Figure 4D, the individual bases that each bar represents is not clear in the x-axis.

4. The authors should consult with an English-speaking colleague to read the manuscript. There are a number of grammatical errors in the text.

5. There have been advances in delivering base editors in vivo by AAV using split-intein systems. These approaches have been used for both SpCas9 editors (Lim et al Mol. Ther. 2020; Levy et al. Nat. Biomed. Eng. 2020) and SaCas9 editors (Villiger, et al. Nat. Med. 2018). The authors specifically should mention these approaches when discussing or reflecting on their MS2-based strategy.

6. Statistics are missing from the manuscript and figures.

**Authors Response**

**Point-by-point responses to the reviewers’ comments:**

Comment

*Response*

Reviewer #1: This manuscript presented by Wang and colleagues described a base editing system through tethering of deaminases with modified sgRNA scaffold via MS2/MCP interaction. The authors first tested the optimal position in sgRNA to insert the MS2 sequence which induced highest editing efficiency through co-transfection of MCP-Apobec1/UGI plasmids. Then this system was expanded to adenine deaminase and demonstrated the possibility of simultaneous A/C base conversion with the same target. The system is interesting, but it is a preliminary study for the characterization of this system, more extensive evaluation and application should be performed.

Major concern:

1. Novelty: Since the strategy has been demonstrated in previous report (Nature Methods, 2016), it is easy to speculate that similar strategy would function. Comparing the data, it seems that the activity of sgBE is higher than CRISPR-X, but direct comparison should be done in this study. Moreover, there are different RNA/RBP systems adapted for CRISPR system, a profound study besides MS2/MCP system should be performed to obtain a optimized system.

*Response: We thank the reviewer for this valuable advice and have compared the activity of sgBE to that of CRISPR-X in 5 target sites. The in-parallel comparison revealed that sgBE was indeed much more efficient than CRISPR-X, which was likely due to the fact that sgBEs nicked the un-edited strand while CRIPSR-X did not. To examine the performance of sgBEs using other RNA/RBP interaction system, we systematically substituted MS2 tag with another popular RNA tag, box-B. The resulting box-B tagged sgBEs generally produced similar editing efficiency and pattern to MS2 tagged sgBEs.*

2. Specificity: Specificity is a critical issue to evaluate the base editing system especially the authors mentioned the potential clinical applications. However, there is no single data to show the sequence dependent or independent off-target effects of the sgBE system. Since this study using a free deaminase overexpression strategy, the sequence independent off-target editing effects should be higher. Analysis of these Cas9 independent off-targets will be critical to demonstrate if these base editors will be therapeutically useful. While whole genome sequencing may be outside the scope of this paper, the authors should at least comment on this potential downside of the technology. However, the sequence dependent off-target effects should be extensively studied. Moreover, RNA off-targeting should be tested to show whether sgBE system increases side effect. Previous study showed AID exhibited very low RNA off-targeting, could the system be optimized and adapted to AID, since the author showed sgBE-AID was not efficient currently.

*Response: We have discussed the sequence independent off-target effects of sgBEs in the revised discussion section. We examined the sequence dependent off-target effects of sgBEs, with a panel of bioinformatically predicted off-target sites, and compared the effects of sgCBEs to those of BE3. We found sgCBEs produced comparable off-target editing to BE3 in the majority of these off-target sites. For RNA off-target editing, we have substituted sgBE-AID with sgBE-Target-AID because Target-AID has been shown to have similar RNA editing activity to, while much higher on-target DNA editing activity than AID. As shown in the revised Fig. 2, Target-AID was indeed stronger than AID, but much weaker than Apobec1 and A3A. Recently, there was growing evidence showing that deaminases, such as Apobec1, A3A and ABE, can be engineered to reduce their RNA editing activity while without significantly affecting on-traget DNA editing activity. The main aim of this work is to engineer sgRNA scaffold to recruit deaminases to different locations of cas9 complex, and thereby to create novel base editors with diversified editing features. In the near future, we will test RNA off-target editing of sgBEs harboring optimized deaminases.*

3. Efficiency: The previous reported CRISPR-X system showed very limited activity. Through test of variant deaminases and using Cas9n instead of dCas9, the seBE system showed much higher activity than CRISPR-X. Thus the increase of efficiency is the shining point, but only a few targets have been investigated making the advantage unconvincing. To analyze the editing efficiency, deep-sequencing analysis should be applied. The EditR program is not accurate for base conversions. Moreover, since BE3 or BE4 and other advanced CBEs have shown very high efficiency and specificity, directly comparing of sgBE with BE3 should be performed for more targets.

*Response: We have added another six targets and a total of 12 targets were used to characterize those sgCBEs. We agree with the reviewer that EditR program is not so accurate as deep sequencing for base conversions, but EditR is rapid, straightforward and inexpensive. For many cases, especially when comparing different base editors in the same target sites, EditR is good enough because it can detect as small as 2.5% difference. Therefore, many labs used this method in their publications. As the reviewer suggested, deep-sequencing has been performed to analyze the editing efficiency in a part of targets in the revised manuscript, which raised similar conclusions to the ones raised by EidtR analysis. In addition, comparing of sgBEs with BE3 have been performed for more targets, all of which contained multiple Cs in the putative editing window (revised Fig.2).*

4. Products purity and indel rates: The products purity and indel rates of sgBE should be examined.

*Response: Deep-sequencing analysis has been conducted to examine the product purity and indel rates of sgBEs (revised Fig S6).*

5. Editing window: The author claimed they were able to diversify the tool-box with a series of pattern-specific editors. It is interesting, but again only few targets have been tested. It is hard to draw any conclusion. Moreover, if it is true, what is the advantage of BEs with wider editing window? The authors should discuss the feature and show the utility.

*Response: Thanks for the reviewer’s advice. The pattern-specific editing of sgBEs had been demonstrated with additional targets. A heat map showing the editing window of each sgCBEs has been made in the revised Fig.2. We have also discussed these features and the possible utility of pattern-specific or wide-window sgBEs in the revised discussion section.*

6. Dual base editors: It is interesting to generate simultaneous A/C mutation, but what is the ratio of single A>G, C>T and simultaneous A/C allele? More targets and deep-sequencing should be performed. Since the authors claimed that (SL1+MS2)+(SL3_MS2) sgRNA showed two peaks, why not using two different scaffolds and RBPs to recruit cytosine and adenine deaminases to achieve simultaneous mutation. The strategy used in this study to create simultaneous A/C mutation seems not a optimal one. Moreover, recent publication by Caixiao Gao's group has shown a new system called STEME to generate simultaneous A/C mutation. It is better to compare these two system.

*Response: Thanks for the review’s good suggestions. The efficiency of dual base editors had been examined with additional targets that contained both A and C in the putative editing window. The ratio of single A>G or C>T and simultaneous A/C conversion allele was analyzed via deep-sequencing, which was shown in the revised Fig.4. We have coupled different cytosine deaminases to ABE7.10, and found that the combination of apobec1 and ABE7.10 produced most efficient simultaneous editing. As the reviewer suggested, we also designed a dual RNA tag sgBE system, by using MS2 and Box-B tag to recruit cytosine and adenine deaminases. Compared to our original strategy, dual tag sgBE produced much lower editing (Figure S12). In addition, we have compared our dual base editor system to STEME in the revised Fig.4.*

7. Utility: This study did not show any application, although they claimed that the system could be easily packaged into AAV. However, as I know not single vector can package sgBEs. Since numerous studies have shown efficient editing of traditional BE3/BE4 to treat genetic diseases through intein-mediated AAV system, only discussion is not enough to show the advantage.

*Response: We thank the reviewer for this suggestion and have packaged saCas9 derived sgCBE into AAV2. Transduction of AAV2-sgCBE produced remarkable base editing in human cells, suggesting that our system is compatible to AAV delivery and thus hopefully will be applied in treating genetic diseases.*

8. From the discussion and the results, the advantages as well as the characteristics of sgBEs were not fully presented.

*Response: The editing features of sgBEs have been reexamined and elaborated in the revised results, and the advantages and possible disadvantages have been discussed in the revised discussion section.*

Reviewer #2: Wang et al develop an approach to recruit cytosine and adenine deaminase catalytic domains to a target site for base editing using sgRNA scaffolds with embedded MS2-targeting domains that bind to a separately co-delivered MS2-deaminase fusion protein. They observe reasonable editing rates and find that incorporating MS2 domains within different sgRNA structural elements can alter the editing window, which is an interesting finding. They show that this approach can enable dual A>G and C>T editing within a single target, though with some caveats related to efficiency.

The authors describe an approach similar to one established Hess et al, though that strategy was primarily focused on driving somatic hypermutation. A prior study also established that adenosine and cytosine deaminase domains could both be fused to a Cas9 scaffold to facilitate dual editing. The authors method is distinct from that approach and could offer the advantage of more flexibility owing to the potential for editing window tuning. This work is interesting and should serve as a valuable editing to the growing base editing toolbox. However, I have some concerns. The authors must expand the number of sites they targeted with some constructs and generate design rules that could help the community capitalize on their findings. Specifically, right now, the results are only observations, and leave much to be desired in terms of forward design and implementation. Additionally, there is insufficient evidence to support some claims, including the application of this approach for AAV.

Major comments:

1. In Fig. 1C, the authors should include the editing frequencies observed using a native cytidine deaminase. These control points appear to be missing from this data and other data throughout the manuscript.

*Response: We thank the reviewer for the suggestion and are very sorry for this negligence. We have analyzed frequencies of base conversion in untreated cells and native Apobec1 treated ones, and as expected we found no obvious base conversion. Because untreated group was the most frequently negative control in base editing, the data from untreated cells were added as negative controls in each figure.*

2. On Pg 5, line 9, the authors say, "editing efficiency in each targetable cytosines by SL+MS2 sgRNA was ~40% higher than that by (SL1+MS2)+(SL3+MS2) sgRNA ( 11.55% VS 16.14%) (Fig. 1c and Additional file 1: Fig. S1)." This sentence is unclear to me. Do they mean the inverse? That (SL1+MS2)+(SL3+MS2) sgRNA was higher than SL+MS2 sgRNA? Also, the percentages do not match the data being discussed. A 40% increase was only observed in Fig. 1C left, which doesn't seem to be the data mentioned in this sentence.

*Response: We thank the reviewer for pointing out this error. The percentages were inversely positioned (should be 16.14% VS 11.55%), which have been corrected in the revised manuscript. These percentages were averages that were calculated from the editing efficiency of each targetable cytosine. Taken site C as an example (revised Fig. 1D), the editing of (SL1+MS2)+(SL3+MS2) sgRNA was similar to or even slightly higher than that of SL4+MS2 sgRNA in positions C5, C12 and C13. However, in C6 and C8, (SL1+MS2)+(SL3+MS2) sgRNA produced much lower editing than SL4+MS2 sgRNA. Therefore, the average value of all targetable cytosines of (SL1+MS2)+(SL3+MS2) sgRNA is lower than that of SL4+MS2 sgRNA.*

3. The authors conclude that SL4-MS2 has a much broader editing window than (SL1+MS2)+(SL3+MS2). I think there is insufficient evidence to fully draw this conclusion. I would like to see the authors target additional sequences and confirm the trend on multiple sites.

*Response: Additional target sites were used to compare the editing window of SL4-MS2 to that of (SL1+MS2)+(SL3+MS2). From the data presented in revised Fig2c and 2d, it is clear that SL4-MS2 has a much broader editing window than (SL1+MS2)+(SL3+MS2). We have removed “SL4-MS2 has a much broader editing window than (SL1+MS2)+(SL3+MS2)” from the 1st section of the result in the revised manuscript, since these revised data occurred later than this sentence. Similar conclusion was summarized in the 2nd section.*

4. In Pg. 5, the authors note that different sgCBEs gave unique editing patterns. They should elaborate more on their findings. Additionally, are there any design principles that can be learned from their results?

*Response: Thanks for the reviewer’s suggestion. We have described more details about the performance of sgCBEs in the revised manuscript, with an emphasis on editing pattern. A heat map showing the editing window of each sgCBEs has been summarized in the revise manuscript, which may serve as a guidance for readers to select sgCBEs suitable for their own target sites. And we have also proposed several strategies for designing multiple functional editing tool in the revised discussion section.*

5. The authors created an SaCas9-based sgCBE construct, which they say demonstrates compatibility with AAV delivery. This is not convincingly established to me. For one, SaCas9 cytidine deaminases still exceed the carrying capacity of a single AAV vector. So it is unclear as to what hurdle the authors are overcoming. Two, they do not demonstrate delivery of the SaCas9-sgCBE by AAV. The authors should at least transduce 293 cells with AAV encoding the system to claim they have developed a translational system.

*Response: Thanks for the reviewer’s advice. SaCas9 base editors, such as sa-BE3 had been packaged into AAVs via protein splicing strategy, which require splitting Cas9 protein and potentially attenuated the editing efficiency. Although total SaCas9-based sgCBE elements exceed the carrying capacity of a single AAV, they can be packaged into two AAV vectors, without splitting saCas9 protein. Importantly, the editing efficiency of our sa-sgCBE is similar to or even higher than that of intact sa-BE3. Therefore, we proposed that sa-sgCBE could serve as an AAV compatible base editing system for in vivo gene therapy. To demonstrate this proposal, we have packaged sa-sgCBE into AAV vectors, and observed extensive base editing in mammalian cells (revised Fig. 5d and 5e).*

Minor comments:

1. In Fig. 1, in addition to the 3D structure showed in panel A, a two-dimensional schematic of sgRNA secondary structure with labeling of its specific structures (stem loops) would be useful (similar to that in Fig. 2b).

*Response: We thank the reviewer for the good advice and have drawn a two-dimensional schematic of sgRNA secondary structure with stem loops indicated in the revised Fig.1.*

2. The authors are interested in developing tools compatible with AAV delivery, thus it would be useful to indicate the size (in kb) of the constructs in the figures (for example, Fig. 1b which shows three different constructs).

*Response: The size of key elements of sgBEs in each construct was indicated in the revised figures.*

3. The axises in the figures need to be better labeled. For example, in Figure 4D, the individual bases that each bar represents is not clear in the x-axis.

*Response: The axis in each figure has been clearly labeled in the revised figures.*

4. The authors should consult with an English-speaking colleague to read the manuscript. There are a number of grammatical errors in the text.

*Response: We are sorry for those grammatical errors and have asked an English-speaking colleague to edit the revised manuscript.*

5. There have been advances in delivering base editors in vivo by AAV using split-intein systems. These approaches have been used for both SpCas9 editors (Lim et al Mol. Ther. 2020; Levy et al. Nat. Biomed. Eng. 2020) and SaCas9 editors (Villiger, et al. Nat. Med. 2018). The authors specifically should mention these approaches when discussing or reflecting on their MS2-based strategy.

*Response: Thanks for the reviewer’s suggestion. We have discussed these approaches in the revised discussion section.*

6. Statistics are missing from the manuscript and figures.

*Response: Statistical analysis had been performed in the revised manuscript.*

**Second round of review**

**Reviewer 1**

The authors have satisfactorily responded to my comments and  validated their data through additional experiments. The manuscript now is suitable to be published in the Genome Biology.

**Reviewer 2**

The authors revised manuscripts addresses the majority of my concerns. I have two remaining comments:

1. I could not find the indel data in Fig. S6. The authors should double check the figure to make sure it's there or clarify why it is difficult to find.

2. What is the reasoning for using bortezomib? The authors should explain the reasoning in the text and discuss that editing was substantially lower without it, though they used a low MOI for the cell culture study.

**Authors Response**

**Point-by-point responses to the reviewers’ comments:**

1. I could not find the indel data in Fig. S6. The authors should double check the figure to make sure it's there or clarify why it is difficult to find.
*Response: We want to thank the reviewer for pointing out this error. The original Fig. S6b showed the indel data. However, the vertical axis of Fig. S6b was mislabeled to ‘percent of total sequencing reads with A/C converted to G/T’, instead of ‘percent of total sequencing reads with indels’. In the revised version, the labeling of vertical axis was corrected to ‘percent of total sequencing reads with indels’.*

2. What is the reasoning for using bortezomib? The authors should explain the reasoning in the text and discuss that editing was substantially lower without it, though they used a low MOI for the cell culture study.
*Response: We have explained the reason for using bortezomib during AAV transduction in the revised results section, and also have discussed the effects of bortezomib on AAV meditated base editing.*
